# A TLR7-nanoparticle adjuvant promotes a broad immune response against heterologous strains of influenza and SARS-CoV-2

**DOI:** 10.1038/s41563-022-01464-2

**Published:** 2023-01-30

**Authors:** Qian Yin, Wei Luo, Vamsee Mallajosyula, Yang Bo, Jing Guo, Jinghang Xie, Meng Sun, Rohit Verma, Chunfeng Li, Christian M. Constantz, Lisa E. Wagar, Jing Li, Elsa Sola, Neha Gupta, Chunlin Wang, Oliver Kask, Xin Chen, Xue Yuan, Nicholas C. Wu, Jianghong Rao, Yueh-hsiu Chien, Jianjun Cheng, Bali Pulendran, Mark M. Davis

**Affiliations:** 1 Institute for Immunity, Transplantation and Infection, School of Medicine, Stanford University, Stanford, CA USA; 2 Department of Materials Science and Engineering, University of Illinois at Urbana-Champaign, Urbana, IL USA; 3 Department of Microbiology and Immunology, School of Medicine, Stanford University, Stanford, CA USA; 4 Molecular Imaging Program at Stanford, Department of Radiology, Stanford University School of Medicine, Stanford, CA USA; 5 Department of Otolaryngology–Head & Neck Surgery, Indiana University School of Medicine, Indianapolis, IN USA; 6 Department of Biochemistry, University of Illinois at Urbana-Champaign, Urbana, IL USA; 7 Department of Chemistry, Stanford University, Stanford, CA USA; 8 Department of Pathology, Stanford University School of Medicine, Stanford, CA USA; 9 The Howard Hughes Medical Institute, Stanford University School of Medicine, Stanford, CA USA; 10 Present Address: Department of Microbiology and Immunology, Indiana University School of Medicine, Indianapolis, IN USA; 11 Present Address: Department of Physiology & Biophysics, University of California, Irvine, Irvine, CA USA

**Keywords:** Adjuvants, Drug delivery

## Abstract

The ideal vaccine against viruses such as influenza and SARS-CoV-2 must provide a robust, durable and broad immune protection against multiple viral variants. However, antibody responses to current vaccines often lack robust cross-reactivity. Here we describe a polymeric Toll-like receptor 7 agonist nanoparticle (TLR7-NP) adjuvant, which enhances lymph node targeting, and leads to persistent activation of immune cells and broad immune responses. When mixed with alum-adsorbed antigens, this TLR7-NP adjuvant elicits cross-reactive antibodies for both dominant and subdominant epitopes and antigen-specific CD8^+^ T-cell responses in mice. This TLR7-NP-adjuvanted influenza subunit vaccine successfully protects mice against viral challenge of a different strain. This strategy also enhances the antibody response to a SARS-CoV-2 subunit vaccine against multiple viral variants that have emerged. Moreover, this TLR7-NP augments antigen-specific responses in human tonsil organoids. Overall, we describe a nanoparticle adjuvant to improve immune responses to viral antigens, with promising implications for developing broadly protective vaccines.

## Abstract (web summary)

A nanoparticle-based adjuvant incorporating a Toll-like receptor 7 agonist elicits cross-reactive antibodies for both dominant and subdominant epitopes and enhances immune responses against multiple variants of influenza and SARS-CoV-2.

## Main

Vaccines represent one of the most efficient and cost-effective means to control dangerous pathogens and preserve public health^1,2^. Yet, many challenges to vaccine development persist. Current influenza vaccines induce antibodies against the immunodominant part of virus—the globular head of haemagglutinin (HA). However, influenza viruses constantly undergo antigenic drift, resulting in limited breadth and inadequate effectiveness of current seasonal influenza vaccines^3^. While not as variable as influenza, many variants of severe acute respiratory syndrome coronavirus 2 (SARS-CoV-2) have been reported^4,5^. These variants in general carry mutations in the receptor-binding domain (RBD)-containing spike protein, which can substantially reduce vaccine effectiveness^6^. As such, there is a critical need to develop broadly protective vaccines that can induce cross-reactive antibody responses against multiple viral variants.

Compared to the immunodominant yet hypervariable head domain of HA, subdominant epitopes in the HA stem are more conserved and have the potential to generate cross-reactive immune responses^7,8^. Tremendous efforts have been focused on antigen design to improve humoral responses against the HA stem region, such as multivalent HA-nanoparticles^9–12^, and ‘headless’ HA-stalk region-based immunogens^13–16^. Although effective, these approaches often involve sophisticated protein engineering processes and may not be rapidly and readily scalable to meet the global demand.

Adjuvants can play a crucial role in modulating vaccine-induced immune responses^17,18^. However, apart from a study ten years ago by Golding and co-workers^19^ that reported that MF59 adjuvant could broaden a flu response, optimizing adjuvants to improve the diversity of a vaccine response has not been extensively studied. Aluminium hydroxide (alum), a primary adjuvant currently used in commercial vaccines^20^, failed to induce broad antibody responses^17^. Agonists of Toll-like receptors (TLRs) have been explored as adjuvant candidates in mice and non-human primates^18,21–27^. Although a library of potent small molecular TLR7 agonists (for example, imidazoquinolines and its derivatives) are under continuous development, their use as vaccine adjuvants has not progressed beyond early clinical trials due to the rapid diffusion from the injection site and subsequent undesirable systemic immune activation^28^. 3M-052 (refs. ^27,29–31^), an imidazoquinoline adjuvant designed with a C18 lipid moiety to slow dissemination from the injection site, was shown to elicit high-magnitude and durable antibody responses in non-human primates^27,29^. Furthermore, an adjuvant system using alum-adsorbed TLR7 agonist (trade name: AS37)^32,33^ has been evaluated in clinical trials^34^. However, using these adjuvants to enhance antibody breadth against mutated influenza viral variants remains largely unexplored.

Here we present an alternative TLR7 agonist nanoparticle (TLR7-NP) adjuvant system with tunable drug loading, narrow size distribution and controlled release kinetics. Compared to the small molecular TLR7 agonist in the mixture with alum (TLR7–alum), TLR7-NP improved in vivo retention, draining lymph node (dLN) accumulation and cellular uptake of TLR7 agonist by various antigen-presenting cells (APCs), leading to persistent activation of dendritic cells (DCs) and B cells in dLNs and significantly enhanced germinal centre (GC) and CD8 T-cell responses. We further demonstrate that TLR7-NP-adjuvanted influenza and SARS-CoV-2 subunit vaccines induced high levels of cross-reactive antibody responses to multiple heterologous viral variants. Remarkably, the TLR7-NP-adjuvanted influenza vaccine induced early and markedly improved titres of subdominant HA-stem specific antibodies and generated effective cross-protection against heterologous influenza viral challenge. We also show that the TLR7-NP adjuvanted a SARS-CoV-2 subunit vaccine with significantly enhanced plasmablast differentiation and antibody isotype switching in a human tonsil organoid system, demonstrating its translational potential.

## Design and characterization of the TLR7-NP

**Fig. 1:**
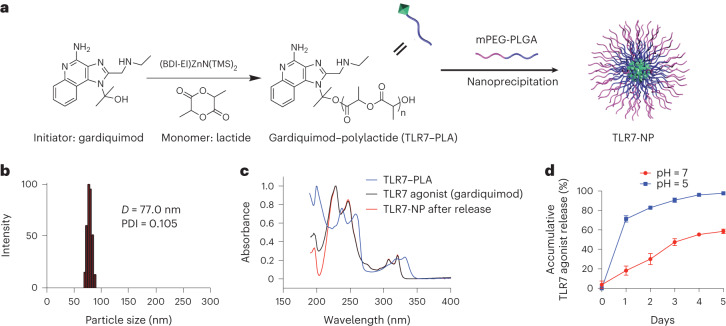
Synthesis, formulation and characterization of TLR7-NP. **a**, Schematic illustration of synthesizing TLR7–PLA polymer conjugate via TLR7 agonist (gardiquimod)-initiated ring-opening polymerization of lactide and preparing TLR7-NP through nanoprecipitation. **b**, Hydrodynamic sizes of TLR7-NP characterized by DLS analysis. *D*, hydrodynamic diameter; PDI, polydispersity index. **c**, The ultraviolet absorbance of TLR7–PLA polymer, gardiquimod and TLR7-NP after release measured by ultraviolet spectrometry at *λ* = 321 nm. **d**, Release kinetic profile of gardiquimod from TLR7-NP in foetal bovine serum (FBS)/phosphate-buffered saline (PBS) (*v*/*v* = 1/1) buffer at pH 5 and pH 7. Data are mean ± s.e.m. with *n* = 3 independent samples for each time point. Source data

**Extended Data Fig. 1:**
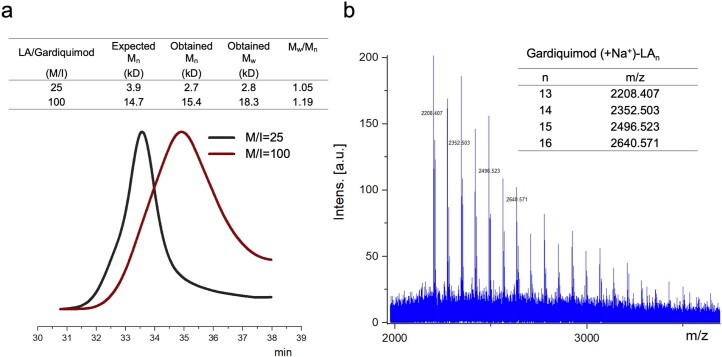
Characterization of synthesized gardiquimod-polylactide (TLR7−PLA) polymer. (**a**) GPC analysis of gardiquimod-LA_n_, equipped with dRI detector. (**b**) MALDI-TOF MS analysis of gardiquimod-LA_n_. The obtained m/z is identical to the calculated m/z of gardiquimod (+Na^+^)-LA_n_ (336.3 + 144.08*n). Matrix:HCCA. Source data

The TLR7-NP was made by co-nanoprecipitation of TLR7–polylactide (TLR7–PLA) polymer conjugates and poly(ethylene glycol)-*b*-poly(lactic-co-glycolic acid) (PEG-PLGA) (Fig. 1a). TLR7–PLA polymer conjugates were synthesized by using gardiquimod, a potent agonist for TLR7 receptor expressed in both mouse and human, to initiate the ring-opening polymerization of lactide^35–37^. This method allowed for quantitative incorporation of gardiquimod into PLA polymers and resulted in TLR7–PLA conjugates with precisely controlled compositions and molecular weights (Extended Data Fig. 1a,b). At a monomer/initiator (LA/gardiquimod) ratio of 25, gardiquimod loading as high as 14.8 wt% was achieved with nearly 100% incorporation efficiency. The resultant TLR7–PLA polymer conjugates mixed with PEG-PLGA and self-assemble into TLR7-NP with 77 nm hydrodynamic diameter and narrow size distributions (polydispersity index = 0.105) characterized by dynamic light scattering (DLS) (Fig. 1b).

In the design of TLR7-NP, gardiquimod was conjugated to PLA polymer through hydrolysable ester bonds. Released gardiquimod from TLR7-NP (red) shared the identical ultraviolet–visible absorbance spectrum as the original gardiquimod (black), providing evidence of releasing unmodified TLR7 agonist without any residual chemical groups (Fig. 1c). To assess the release profile of gardiquimod from TLR7-NP under different physiological conditions, we conducted the release kinetic studies at pH 5 and 7. As shown in Fig. 1d, gardiquimod was released from TLR7-NP in a sustained manner without burst release effects at pH 7, potentially minimizing the undesired systemic diffusion during the circulation. The release rate of gardiquimod from TLR7-NP was accelerated at pH 5, probably due to faster hydrolysis with increased acidities, which is important for robust activation of intracellular TLR7 receptors once internalized into the endosome/lysosome of immune cells (Fig. 1d).

## Lymph node targeting and cellular internalization

**Fig. 2:**
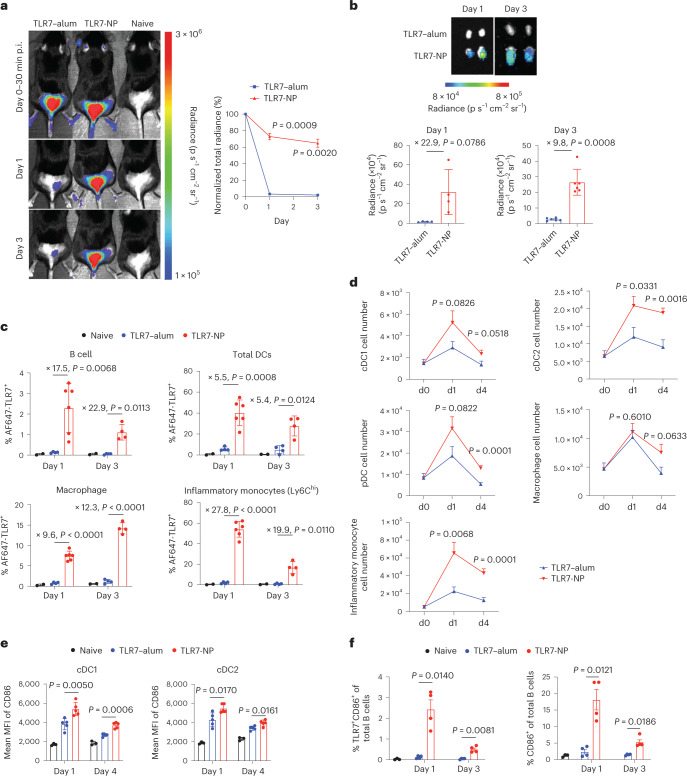
TLR7-NP improves the in vivo performance of gardiquimod and induces persistent activation of antigen-presenting cells in dLNs. C57BL/6 mice were immunized with TLR7–alum- or TLR7-NP-adjuvanted alum-adsorbed OVA (50 μg) at day 0. In **a**,**b**,**c**,**f**, gardiquimod was labelled by AF647 (excitation/emission = 651/672 nm) fluorophore, and an equivalent dose of AF647-gardiquimod (20 nmol) was used for TLR–alum and TLR7-NP. In **d**,**e**, an equivalent amount of gardiquimod (20 μg) was used for both TLR7–alum and TLR7-NP. **a**, Representative whole-body fluorescence imaging and average normalized total radiance from groups of mice (*n* = 3 mice per group) at the injection site over time. p.i., post-injection. **b**, Fluorescence imaging of excised dLNs and plotted integrated mean fluorescence intensity (MFI) at day 1 and 3 (*n* = 4 and 6 LNs per group). **c**, Uptake of AF647-gardiquimod by different APCs in dLNs at day 1 and 3 post-immunization (*n* = 2 and 2 mice for naive, *n* = 4 and 4 mice for TLR7–alum, *n* = 6 and 4 mice for TLR7-NP). Data in **a**–**c** are mean ± s.d. **d**, Number of different innate immune cells at 0 (*n* = 9 naive mice), 1 and 4 days post-immunization (*n* = 9 and 9 mice per group). **e**, The MFI of CD86 expression on cDC1 and cDC2 in dLNs for day 1 and 4 (*n* = 3 and 3 mice for naive, *n* = 5 and 5 mice for TLR7–alum or TLR7-NP). **f**, B-cell uptake and activation was measured by flow cytometry on dLNs on day 1 and 3 (*n* = 4 and 4 mice for TLR7–alum, *n* = 4 and 4 mice for TLR7-NP). Data in **d**–**f** are mean ± s.e.m. All the data are analysed by two-sided, unpaired *t*-test with Welch’s correction. *P* values are as shown. Data were combined from two independent experiments (**b**,**c**,**d**,**f**) or from one representative of two independent experiments (**a**,**e**). Source data

**Extended Data Fig. 2:**
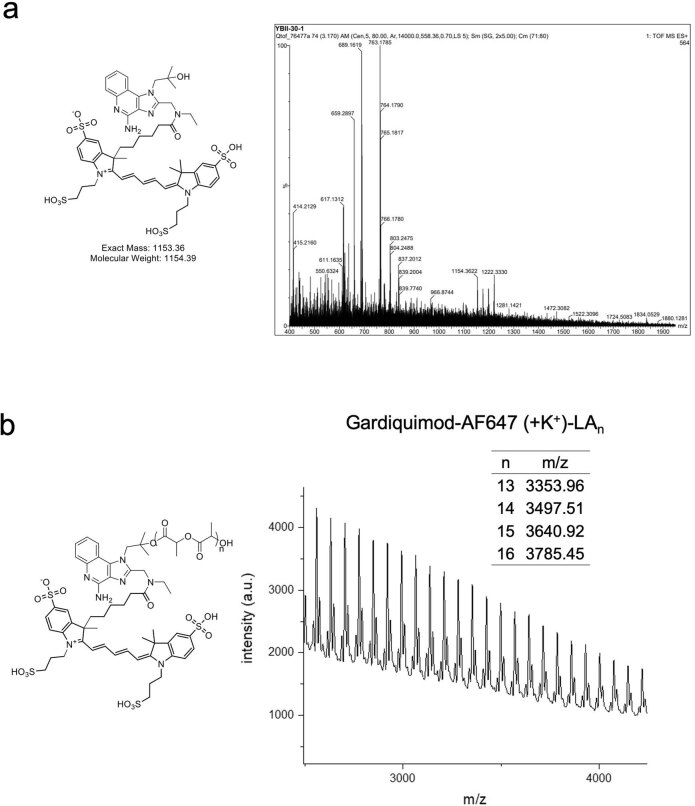
Characterization of AF647-gardiquimod and AF647-gardiquimod (TLR7)-PLA polymer. (**a**) ESI-MS analysis of AF647-gardiquimod. Observed 1154.36 M+H^+^ (**b**) MALDI-TOF MS analysis of AF647-TLR7-LA_n_. The obtained m/z is identical to the calculated m/z of AF647-gardiquimod (+K^+^)-LA_n_ (1192.36 + 144.04*n). Matrix:DMB.

To determine how TLR7-NP influenced the in vivo trafficking of gardiquimod, we labelled gardiquimod in the TLR7–PLA polymer with Alexa Fluor (AF) 647 to prepare AF647-labelled TLR7-NP. For comparison, we mixed AF647-conjugated gardiquimod with alum to make AF647-labelled TLR7–alum (Extended Data Fig. 2a,b). Unlike AS37 formulated by the adsorption of benzonaphthyridine-phosphonates onto alum via phosphonate ligand exchange, gardiquimod in the TLR7–alum is only loosely associated with alum, with an average of 98.4% presenting as free small molecules. For antigen, we used alum-adsorbed protein for all of the studies in the work presented here. AF647-labelled TLR7-NP or TLR7–alum combined with chicken ovalbumin (OVA) was injected subcutaneously into C57BL/6 mice at tail base and tracked by whole-body fluorescence imaging. TLR7–alum was rapidly cleared from the injection site only 1 day post-injection, whereas TLR7-NP persisted for at least 3 days (Fig. 2a). The fluorescence intensity of AF647 in the dLNs was significantly enhanced with TLR7-NP as early as 1 day post-injection and maintained for as long as 3 days post-immunization (Fig. 2b). Compared to TLR7–alum, TLR7-NP fluorescence persisted in dLNs with a 22.9-fold increase at day 1 and a ~10-fold increase at day 3 after immunization. These results indicate that TLR7-NP preferentially targeted and was retained in the dLNs much longer than TLR7–alum.

**Extended Data Fig. 3:**
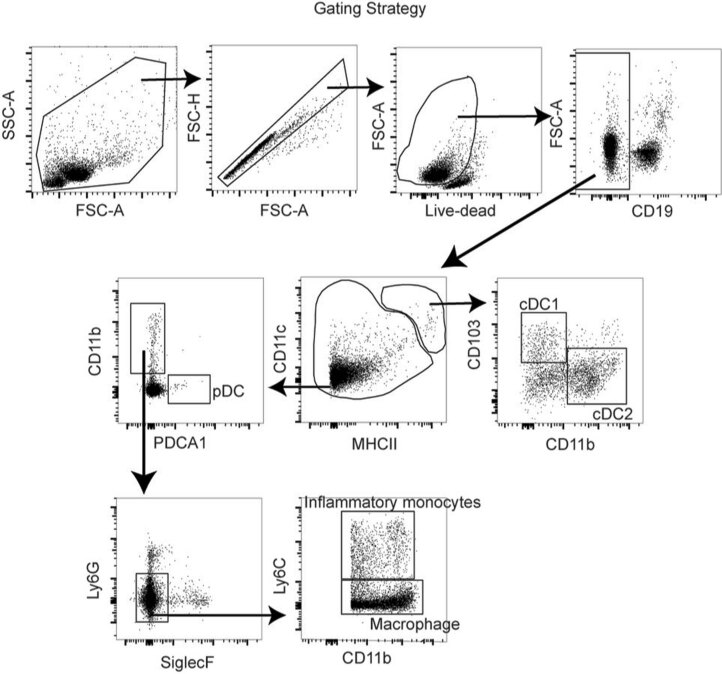
Gating strategy for flow cytometry analysis of DCs and cells of monocytic lineage. Representative flow plots of mouse lymph node samples gated for single cells, live cells, cDC1, cDC2, pDC, inflammatory monocytes, and macrophage cells.

**Extended Data Fig. 4:**
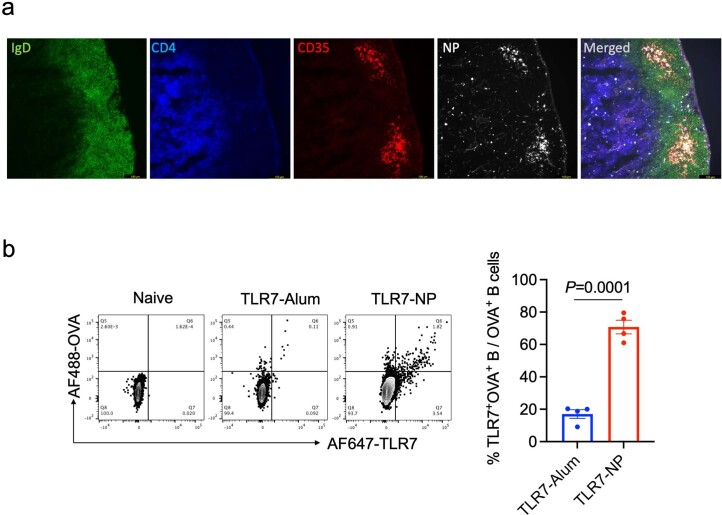
TLR7-NP deposited to B cell follicles and was internalized into antigen-specific B cells. (**a**) Immunofluorescence staining of dLNs 2 days post immunization. B cell zone, T cell zone and FDC network were stained with IgD, CD4 and CD35 antibodies, respectively. AF647-labelled TLR7-NP is shown in white colour. Tissue sections were collected from two biologically independent samples. (**b**) C57BL/6 mice (n = 4 mice per group) were immunized with AF488-labelled OVA (50 μg) plus AF647-labelled gardiquimod (AF647-TLR7, 20 nmol) in either TLR7-Alum or TLR7-NP. The cellular internalization of OVA and TLR7 by B cells from dLNs were measured by flow cytometry 24 h post immunization. Shown are representative flow plots (gated on single live B cells) and the corresponding quantification of TLR7^+^OVA^+^ B cells versus total OVA^+^ B cells. Data are mean ± SEM, P value is determined by two-sided, unpaired t test with Welch’s correction.

Compared to TLR7–alum, TLR7-NP showed a 5.5- to 27.8-fold increase in the cellular uptake of gardiquimod in the vast majority of APCs in dLNs, including B cells, DCs, macrophages and Ly6C^hi^ inflammatory monocytes, as early as 1 day post-injection and persisted for at least 3 days (Fig. 2c). The enhanced cellular uptake was associated with increased cell numbers and persistent activation of DCs and cells of monocytic lineage in the dLNs (Fig. 2d,e and Extended Data Fig. 3). Interestingly, TLR7-NP efficiently accumulated around the follicular dendritic cell (FDC) network in the B-cell follicles 2 days post-immunization (Extended Data Fig. 4a). In line with this, TLR7-NP promoted the uptake of gardiquimod by B cells and induced sustained B-cell activation, characterized by activation marker CD86 at days 1 and 3 post-injection (Fig. 2f). To further examine whether the antigen co-localizes with adjuvant after injection, we labelled the OVA antigen with AF488 and gardiquimod with AF647, respectively, in the TLR7–alum- or TLR7-NP-adjuvanted vaccines. Strikingly, TLR7-NP immunization significantly increased the percentage of B cells that co-internalized the OVA antigen and gardiquimod compared with the TLR7–alum-adjuvanted one (Extended Data Fig. 4b), demonstrating the advantages of TLR7-NP over TLR7–alum in promoting TLR7 activation of antigen-specific B cells.

## GC and extrafollicular B-cell responses

**Fig. 3:**
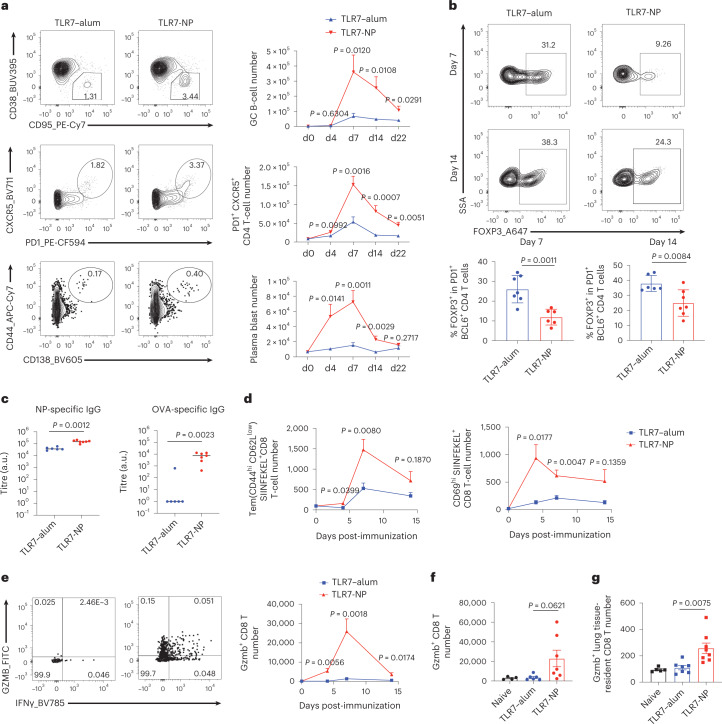
TLR7-NP enhances the magnitude and quality of both humoral and CD8^+^ T-cell responses. Mice were immunized with alum-adsorbed NP-OVA (50 μg) plus gardiquimod (20 μg) in either TLR7–alum or TLR7-NP on day 0. **a**, Representative flow cytometry plots on day 7 and the quantification of immune cells in dLNs from day 4, 7, 14, 22 (*n* = 8, 8, 8, 8 mice for TLR7–alum, *n* = 7, 6, 7, 6 mice for TLR7-NP). **b**, Representative flow cytometry plots and the analysis of Tfr within follicular CD4^+^ T cells (*n* = 7, 6 mice for TLR7–alum, *n* = 6, 7 mice for TLR7-NP). **c**, ELISA analysis of day 14 serum antibodies specific for NP and OVA (*n* = 6, 7 mice for TLR7–alum, TLR7-NP). Data are medians with each dot representing one mouse. *P* values were calculated by Mann–Whitney test. **d**, The quantification of tetramer^+^ effector memory (CD44^hi^ CD62L^low^) and tetramer^+^-activated (CD69^hi^) CD8^+^ T cells on day 4, 7, 14 (*n* = 3 mice for naïve (day 0), *n* = 4, 6, 5 mice for TLR7–alum, *n* = 4, 6, 6 mice for TLR7-NP). **e**, Representative flow cytometry plots and the quantification of Gzmb^+^ CD8^+^ T cells in dLNs on day 4, 7, 14 (*n* = 5, 7, 6 mice for TLR7–alum, *n* = 5, 6, 7 mice for TLR7-NP). **f**, Total number of Gzmb^+^ CD8^+^ T cells in the lungs on day 14 (*n* = 6, 7 mice for TLR7–alum, TLR7-NP). **g**, Total number of Gzmb^+^ lung tissue-resident CD8^+^ T cells on day 21 (*n* = 7, 8 mice for TLR7–alum, TLR7-NP). Except for the data in **c**, all other data are mean ± s.e.m. The data in **a**,**d**,**e** are analysed by two-sided, unpaired *t*-test; the other data are analysed by two-sided, unpaired *t*-test with Welch’s correction. Data in **a**,**e** and **b**,**c**,**d**,**f**,**g** were combined from three and two independent experiments, respectively. Source data

**Extended Data Fig. 5:**
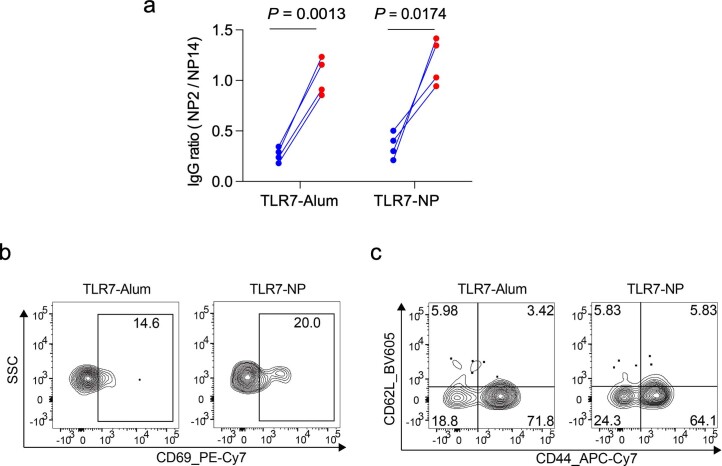
TLR7-NP promoted humoral and cellular immune responses. C57BL/6 mice were immunized with Alum-adsorbed NP-OVA (50 μg) plus gardiquimod (20 μg) in either TLR7-Alum or TLR7-NPs on Day 0. (**a**) Serum samples from immunized mice (n = 4 mice per group) collected 2 weeks and 3 weeks post immunization were analysed by ELISA for the ratio of IgG antibodies binding to NP2-BSA and NP14-BSA. P values are determined by two-sided, paired t test. (**b**, **c**) Representative flow cytometry plots of antigen-specific activated (CD69^hi^) and effector memory (CD44^hi^ CD62L^lo^) CD8^+^ T cells and on Day 7. Cells are gated on SIINFEKEL-tetramer^+^ CD8^+^ T cells.

The enhanced and sustained activation of innate immune cells and B cells by TLR7-NP encouraged us to assess the subsequent effects on humoral immune responses, which largely depend on GC reactions^38^. To test whether TLR7-NP can alter GC dynamics, we immunized mice with TLR7-NP- or TLR7–alum-adjuvanted NP-OVA and performed a time-course study from day 4 to day 22 post-immunization. As shown in Fig. 3a, compared with TLR7–alum, TLR7-NP-adjuvanted antigen immunization induced a strikingly high level of GC B-cell responses in dLNs. Consistent with GC B-cell kinetics, CXCR5^+^ PD1^+^ follicular CD4^+^ T cells were also highly expanded in TLR7-NP-immunized mice. These follicular CD4^+^ T cells contain T follicular helper cells (Tfh) and T follicular regulatory cells (Tfr). Tfh provide essential help to GC B cells while Tfr restrains GC reaction^39^. Interestingly, the percentage of Tfr in TLR7-NP-immunized mice is significantly lower than that in TLR7–alum-immunized mice (Fig. 3b). All these data strongly suggest that TLR7-NP could potently promote the GC response. Interestingly, TLR7-NP could also promote the extrafollicular B-cell response with markedly increased numbers of early plasmablasts (Fig. 3a). Mice immunized with TLR7-NP- or TLR7–alum-adjuvanted NP-OVA showed similar affinity maturation as probed by the ratio of high-affinity IgG (NP2-BSA binding) versus total IgG (NP14-BSA binding) from week 2 to week 5 post-immunization (Extended Data Fig. 5a). Strikingly, mice immunized with TLR7-NP, but not TLR7–alum, can generate high levels of antibodies against the subdominant OVA epitopes at day 14 post-immunization, suggesting the change of immunodominance (Fig. 3c).

## Antigen-specific CD8^+^ T-cell responses

TLR7-NP has efficiently induced persistent mobilization and activation of conventional type 1 dendritic cells (cDC1), which are vital for cross-priming CD8^+^ T cells. Indeed, we found that TLR7-NP-adjuvanted vaccine significantly induced early activation of antigen-specific CD8^+^ T cells (CD69^hi^ SIINFEKEL^+^) at day 4 post-immunization, and dramatically increased the numbers of CD44^hi^ CD62L^low^ effector memory CD8^+^ T cells (Tem) at day 7 (Fig. 3d and Extended Data Fig. 5b). This change in Tem is due to the increased total numbers of antigen-specific CD8^+^ T cells, because the percentage of CD44^hi^ CD62L^low^ CD8^+^ T cells within SIINFEKEL^+^ T cells was not different between the TLR7-NP and TLR–alum groups (Extended Data Fig. 5c). TLR7-NP also efficiently induced functional Granzyme-producing (Gzmb^+^) CD8^+^ T cells compared to the TLR7–alum immunization (Fig. 3e). Furthermore, TLR7-NP but not TLR7–alum immunization induced Gzmb^+^ CD8^+^ T cells in the lungs of the mice (Fig. 3f). We also confirmed that TLR7-NP led to a significant increase of Gzmb-producing, lung-tissue-resident CD8^+^ T cells compared with TLR7–alum at day 21 post-immunization (Fig. 3g). Altogether, TLR7-NP is superior to TLR–alum in inducing potent CD8^+^ T-cell responses, which play a crucial role in killing intracellular pathogen-infected cells to control viral replication.

## Cross-reactive and stem-specific antibody responses

**Fig. 4:**
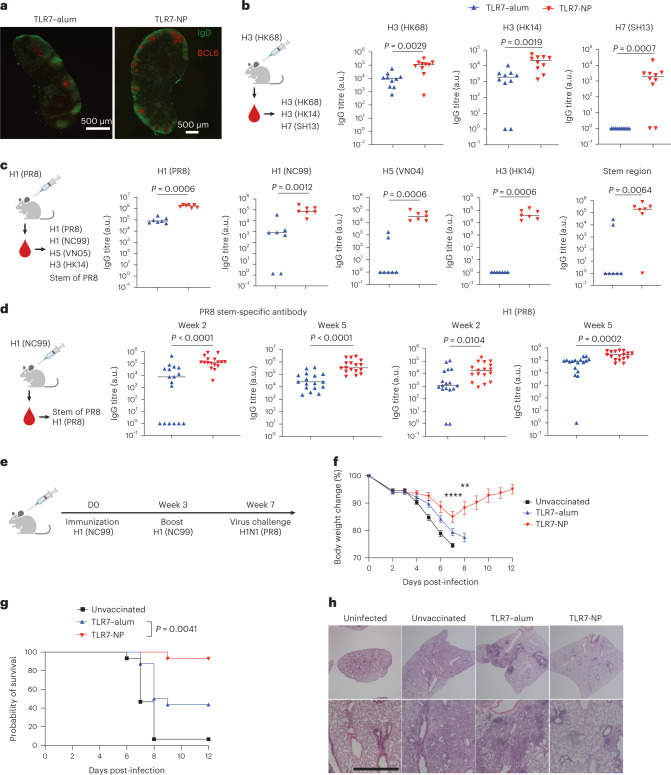
TLR7-NP-adjuvanted HA immunization elicits cross-reactive antibody responses and protects the mice from lethal heterologous viral challenge. **a**, Mice were immunized with alum-adsorbed PR8 HA (10 μg) plus gardiquimod (20 μg) in either TLR7–alum or TLR7-NP at day 0. The GC response in the dLNs (day 10) was analysed by immunofluorescence. The B-cell zone and GC were stained with IgD (green) and BCL6 (red), respectively. Data show one representative LN image from two mice of each group. **b**–**d**, Mice were immunized with alum-adsorbed HA (10 μg, strain as indicated) adjuvanted by gardiquimod (20 μg) in TLR7–alum or TLR7-NP. Serum samples collected 2 weeks (**b**–**d**) and 5 weeks (**d**) post-immunization were analysed by ELISA for antibodies binding to HAs from homo- and heterologous strains as indicated (**b**–**d**) and to the stem region of PR8 HA (**c**,**d**). All the data are medians with each dot representing one mouse (*n* = 10, 7, 17 mice per group in **b**–**d**). *P* values were calculated by the Mann–Whitney test. **e**, Schematic illustration of the experimental design. **f**,**g**, The body weight changes (**f**) and survival (**g**) of mice (*n* = 15, 16, 14 mice for naive, TLR7–alum, TLR7-NP). The data in **f** are mean ± s.e.m. and are analysed by two-way analysis of variance with Sidak’s multiple-comparisons test between the TLR7–alum and TLR7-NP groups (****P < 0.0001, on day 7; **P = 0.0071, on day 8). Data in **g** are analysed by the log-rank (Mantel–Cox) test. **h**, Histological examination of the lungs from different groups of mice 14 days post-infection (top 2×, bottom 8×; scale bar, 1,000 μm). Data show one representative of at least four mice from each group. Data were combined from two independent experiments in **b**,**d**,**f**,**g** or from one representative of two independent experiments in **c**. Source data

**Extended Data Fig. 6:**
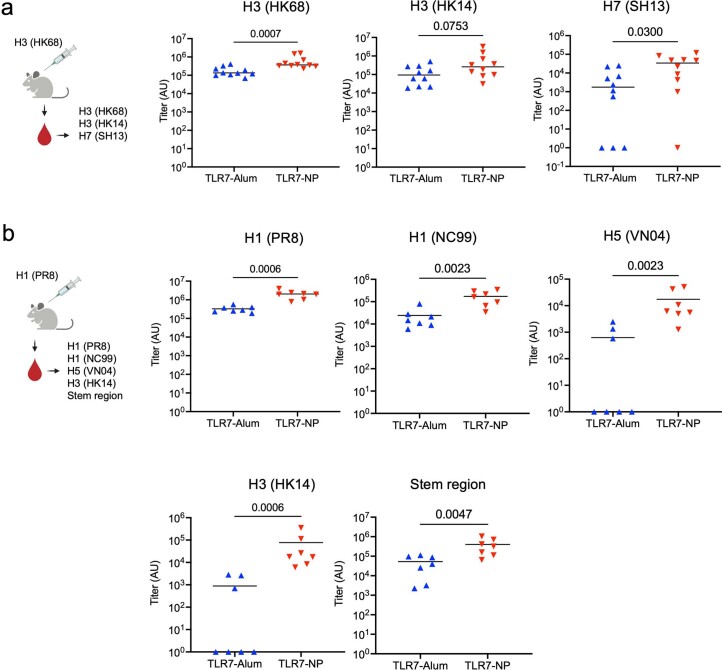
TLR7-NP adjuvanted HA vaccination elicited cross-reactive antibodies. C57BL/6 mice (n = 10 mice per group in a, n = 7 mice per group in b) were immunized with Alum-adsorbed HA (10 μg, strain as indicated in a and b) plus gardiquimod (20 μg) in TLR7-Alum or TLR7-NP. Serum samples collected five weeks post immunization were analysed by ELISA for antibodies binding to HAs from homo- and heterologous strains as indicated. All the data are medians with each dot representing one mouse. *P* values were calculated by Mann-Whitney Test.

**Extended Data Fig. 7:**
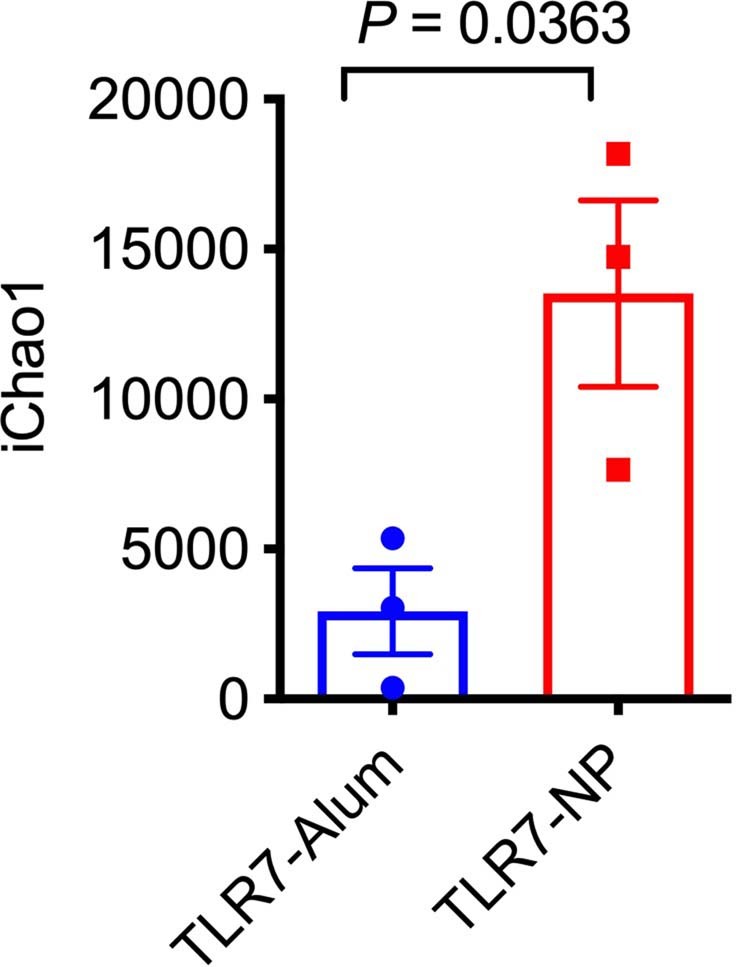
Comparing clonal diversity of GC B cells from TLR7-Alum and TLR7-NP immunized mice. C57BL/6 mice (n = 3 per group) were immunized with Alum-adsorbed PR8-HA (10 μg) adjuvanted by gardiquimod (20 μg) in TLR7-Alum or TLR7-NP. BCRs of GC B cells sorted from the draining LNs14 days post immunization were sequenced by mouse IGH assay. Bar graph showing iChao1 index of BCRs. Data are mean ± SEM. P value is determined using two-sided, unpaired student t-test.

The potent effect of TLR7-NP on promoting GC responses and increasing the Tfh/Tfr ratio suggests its potential to support more diversified B-cell clones in the GCs to increase the breadth of antibody response against influenza HA. To test this possibility, we first performed the immunofluorescence staining of dLNs from mice immunized with TLR7-NP- or TLR7–alum-adjuvanted HA. The results showed that TLR7-NP induced more GCs 10 days post-immunization (Fig. 4a). We next immunized mice with TLR7-NP- or TLR7–alum-adjuvanted H3 HA from influenza strain A/Hong Kong/1/1968 (HK68), a group 2 influenza viral variant, and examined antibodies against HAs from heterologous strains at week 2 and week 5 post-immunization (Fig. 4b and Extended Data Fig. 6a). The results showed that TLR7-NP increased antibody titres against not only the H3 HA from HK68, but also the HA from another H3N2 strain, A/Hong Kong/4801/2014 (HK14). Remarkably, 80% of mice immunized with TLR7-NP-adjuvanted HK68 HA generated antibodies at week 2 against HA from H7N9 A/Shanghai/02/2013 (SH13), a highly pathogenic influenza viral variant from a different subtype. In contrast, these heterosubtypic antibodies could not be detected in the mice immunized with TLR7–alum-adjuvanted HA (Fig. 4b). We then performed a similar experiment but used group 1 HA from H1N1 A/Puerto Rico/8 (PR8) virus as immunogen. We found that TLR7-NP-adjuvanted PR8 HA also elicited significantly higher levels of antibodies against HAs of cross-subtype or even cross-group compared with the TLR7–alum-adjuvanted one (Fig. 4c and Extended Data Fig. 6b). Importantly, in contrast to TLR7–alum, TLR7-NP can promote the generation of antibodies against the conserved yet subdominant stem region (Fig. 4c). With the change of immunogen from another strain NC99 H1 HA, TLR7-NP was still able to enhance antibody responses towards the stem region as well as heterologous PR8 HA (Fig. 4d). Hence, using three different HAs as the immunogen, we demonstrated the potential of TLR7-NP as an adjuvant to overcome immunodominance to induce cross-reactive antibody responses. To further explore how TLR7-NP may affect the B-cell repertoire in GC, we sorted GC B cells from dLNs of mice 14 days post-immunization and sequenced the IgH of their BCRs. We found that BCRs from TLR7-NP elicited GCs that had a significantly higher iChao1 index than the TLR7–alum-induced ones, suggesting a more diverse BCR repertoire (Extended Data Fig. 7).

## Protection against heterologous influenza viral challenge

**Extended Data Fig. 8:**
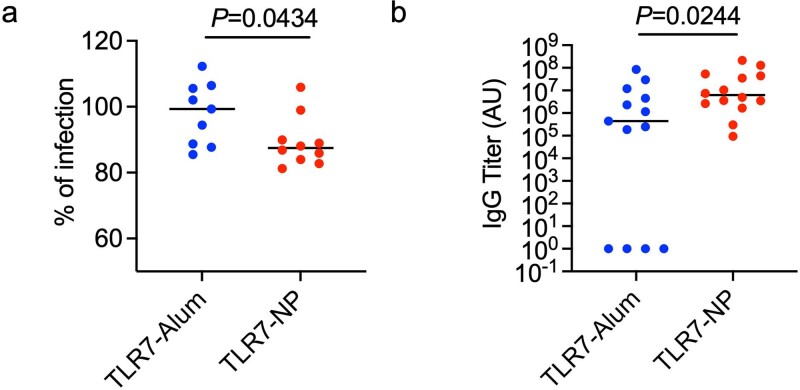
Serum analysis of mice immunized with TLR7-NP or TLR7-Alum adjuvanted HA. (**a**) Neutralization ability of H1N1 A/Puerto Rico/8/34 virus by sera from mice vaccinated with Alum-adsorbed HA of H1HA (NC99) (10 μg) plus gardiquimod (20 μg) in either TLR7-Alum (n = 9 mice) or TLR7-NP (n = 10 mice) at week 5 post immunization. All the data are medians with each dot representing one mouse. *P* values were calculated by two-sided, unpaired t test with Welch’s correction. (**b**) Mice were immunized with Alum-adsorbed H1HA (NC99) (10 μg) adjuvanted by gardiquimod (20 μg) in TLR7-Alum or TLR7-NP. Serum samples (n = 13 mice from TLR7-Alum group, n = 14 mice from TLR7-NP group) collected 14 days post viral challenge of H1N1 A/Puerto Rico/8/34 virus were analysed by ELISA for antibodies binding to stem region of PR8 HA. All the data are medians with each dot representing one mouse. *P* values were calculated by Mann-Whitney Test.

To evaluate the capability of TLR7-NP to induce cross-protection against heterologous influenza viruses, we vaccinated mice with TLR7-NP- or TLR7–alum-adjuvanted NC99 HA at week 0 and week 3, and challenged mice with a lethal dose of PR8 H1N1 virus (a heterologous strain) at week 7 (Fig. 4e). In line with the observation of enhanced cross-reactivity and heterologous viral neutralizing ability (Fig. 4d and Extended Data Fig. 8a), TLR7-NP outperformed TLR7–alum at protecting mice from body weight loss upon heterologous viral challenge (Fig. 4f). Mice vaccinated with TLR7–alum-adjuvanted NC99 HA failed to develop effective cross-protection against PR8 H1N1 viruses as only 43.8% of the mice survived the challenge (Fig. 4g). In contrast, 92.9% of mice vaccinated with TLR7-NP-adjuvanted HA fully recovered after infection (Fig. 4g). Histological analysis further demonstrated that TLR7-NP-adjuvanted HA vaccination could fully protect the mice from infection-induced pulmonary damage (Fig. 4h). In addition, mice that received the TLR7-NP-adjuvanted vaccine had significantly higher levels of HA-stem-specific antibodies 14 days after live viral challenge compared with the mice in the TLR7–alum group (Extended Data Fig. 8b).

## Cross-reactive antibodies against SARS-CoV-2 variants

**Fig. 5:**
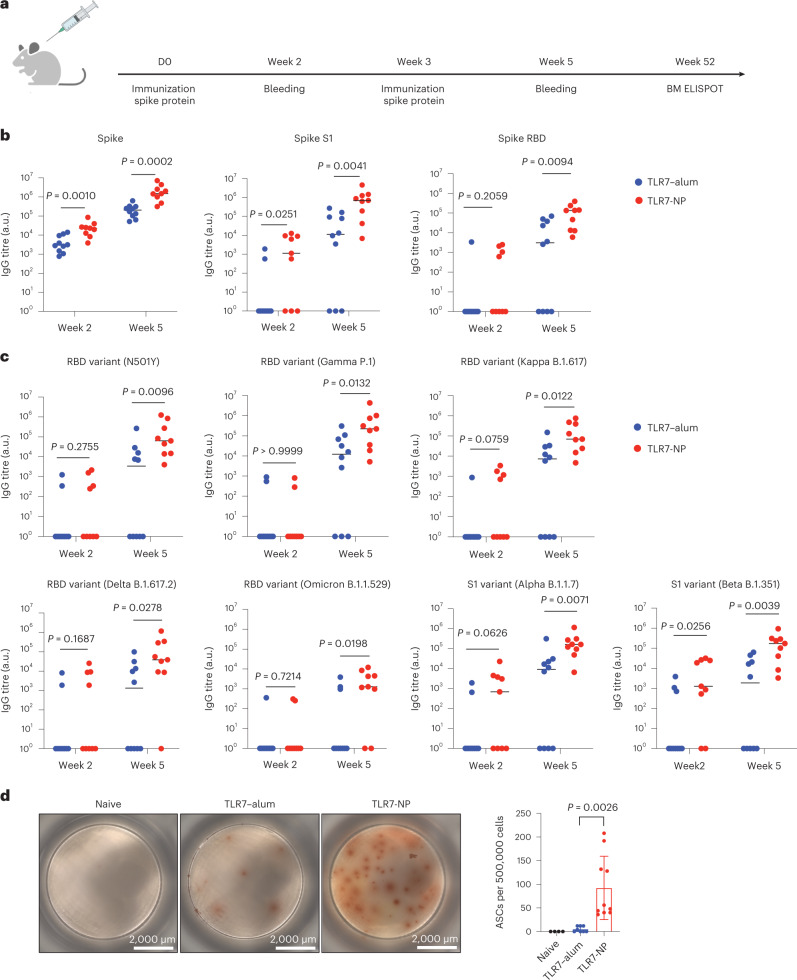
TLR7-NP-adjuvanted SARS-CoV-2 spike immunization induces cross-reactive antibodies against multiple viral variants. **a**, C57BL/6 mice were immunized with the alum-adsorbed full-length SARS-CoV-2 spike protein (5 μg) plus gardiquimod (20 μg) in either TLR7–alum or TLR7-NP at day 0. **b**,**c**, Serum samples were collected on week 2 and week 5 and analysed by ELISA for antibodies binding to Spike RBD, Spike S1 and Spike (wild-type strain) (**b**) and RBD or S1 from variants of concern (**c**). All the data are medians with each dot representing one mouse (*n* = 10, 9 mice for TLR–alum, TLR7-NP). Data are analysed by Mann–Whitney test. *P* values are as shown. **d**, TLR7-NP-adjuvanted spike vaccine in comparison with TLR7–alum-adjuvanted spike vaccine induces significantly higher spike-specific antibody-secreting cells (ASCs) in the bone marrow. Scanned ELISPOT plate images of ASCs at 1 year post-immunization assayed in bone marrow aspirate are shown (left). Quantification of the frequencies of IgG-secreting Spike-specific ASCs in bone marrow aspirates (right) (*n* = 2, 4, 5 mice for naive, TLR7–alum, TLR7-NP; two samples from each mouse). Data are analysed by unpaired *t*-test with Welch’s correction. *P* values are as shown. Data were combined from two independent experiments. Source data

The success of the influenza vaccine prompted us to explore whether TLR7-NP adjuvant could also help increase the breadth of antibody responses against SARS-CoV-2 variants. We immunized C57BL/6 mice with TLR7–alum- or TLR7-NP-adjuvanted SARS-CoV-2 spike protein from the wild-type virus (Fig. 5a) and characterized antibody responses against the wild-type RBD, spike and S1 protein, and against RBD and S1 protein from several variants of concern (Fig. 5b,c). After two immunizations, all mice immunized with TLR7-NP-adjuvanted spike developed high titres of antibodies against RBD with the N501Y mutation and RBD of the Gamma variant (P.1) and the Kappa variant (B.1.617), and against S1 protein from the Alpha (B.1.1.7) and the Beta (B.1.351) variants. In contrast, mice immunized with TLR7–alum-adjuvanted spike protein developed significantly lower antibody responses against the immunized wild-type spike protein and partial (50–60% of vaccinated mice) responses against these variants. Similarly, for two highly contagious SARS-CoV-2 variants, the Delta variant (B.1.617.2) and the Omicron variant (B.1.1.529), TLR7-NP was able to induce high titres of antibodies against its RBD domain in 77–89% of vaccinated mice, whereas only 30–50% of mice immunized with TLR7–alum-adjuvanted spike protein generated 1–2 log fold lower antibody titres (Fig. 5c). Moreover, the ELISPOT assay showed that TLR7-NP significantly increased the generation of antigen-specific bone marrow long-lived plasma cells compared to TLR7–alum 1 year post-immunization, demonstrating the long duration of humoral memory response (Fig. 5d).

## B-cell responses in three-dimensional human tonsil organoid cultures

**Fig. 6:**
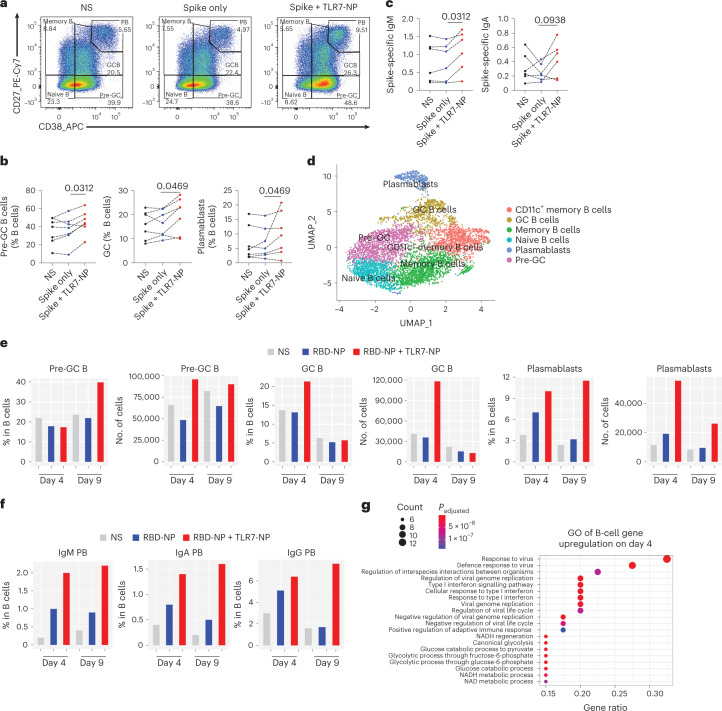
TLR7-NP-adjuvanted SARS-CoV-2 spike protein promotes B-cell differentiation and antibody response in human tonsil organoid cultures. **a**, Representative flow cytometry staining of B-cell phenotypes in unstimulated (NS), full-length spike-protein-only stimulated, and spike protein plus TLR7-NP-stimulated organoid cultures from one donor on day 14. Cells shown are pre-gated on total live B cells (CD45^+^CD19^+^CD3^−^). **b**, Quantification of B-cell differentiation towards pre-GC (CD38^+^CD27^−^), GC (CD38^+^CD27^+^) and plasmablast (CD38^+^CD27^++^) in NS, spike-only stimulated, and spike plus TLR7-NP-stimulated organoid cultures (*n* = 7 donors). **c**, Quantification of spike-specific IgM and IgA from NS, spike-only stimulated and spike plus TLR7-NP-stimulated culture supernatants on day 14 (*n* = 6 donors). *P* values shown were calculated by Wilcoxon matched-pairs signed rank test. **d**, UMAP projection of tonsillar B-cell scRNA-seq clusters. **e**, Quantification of B-cell differentiation subclusters (both frequency and total cell number in organoids) in scRNA-seq. **f**, Frequency quantification of plasmablast subclusters in scRNA-seq. **g**, Gene ontologies (GO) for genes significantly upregulated in TLR7-NP plus antigen stimulated cultures versus antigen-only stimulated cultures on day 4. The top 20 GO terms are ranked by the adjusted *P* values. Source data

To further investigate the translational potential of TLR7-NP adjuvant, we combined it with SARS-CoV-2 spike protein in human tonsil organoid (TOS) cultures, using protein alone as a control^40^. After a 14 day culture, we found TLR7-NP-adjuvanted Spike could significantly promote Pre-GC B, GC B and plasmablast differentiation with enhanced spike-specific IgM and IgA antibody production compared with Spike alone in a subset of donors (Fig. 6a–c). We also tested the combination of TLR7-NP with a clinically tested subunit vaccine, a SARS-CoV-2 spike protein RBD nanoparticle (termed RBD-NP)^41^, in TOS. We performed a time-course study using single-cell RNA sequencing (scRNA-seq) for sorted B cells from unadjuvanted RBD-NP and TLR7-NP-adjuvanted RBD-NP TOS. Six distinct B-cell populations were identified by uniform manifold approximation and projection (UMAP) based on their gene expression profile (Fig. 6d). Both the frequencies and number of GC B cells and plasmablasts were notably increased as early as 4 days post-stimulation with TLR7-NP-adjuvanted RBD-NP compared with RBD-NP alone or non-stimulation (NS) (Fig. 6e), consistent with our mouse studies (Fig. 3). Importantly, TLR7-NP substantially increased IgM, IgA and IgG plasmablast formation in TOS, suggesting its broad effects on the antibody isotype switching of human B cells (Fig. 6f). Gene ontology (GO) analysis further indicated that the genes associated with responses to viruses and type 1 interferon were remarkedly upregulated on 4-day-old TOS stimulated with TLR7-NP-adjuvanted RBD-NP compared to RBD-NP alone (Fig. 6g).

**Extended Data Fig. 9:**
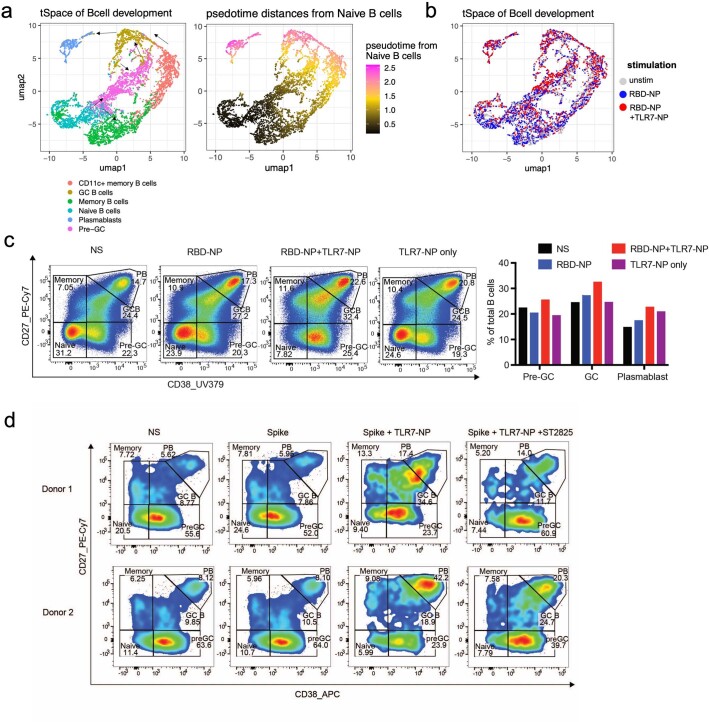
TLR7-NP adjuvanted SARS-CoV-2 subunit vaccine in human tonsil organoids. (**a**, **b**) Trajectory space (tSpace) projections of all B cell differentiation lineages from unstimulated (NS), RBD-NP only stimulated, and RBD-NP plus TLR7-NP stimulated tonsil organoids. Data was collected by one single BD Rhapsody single-cell targeted RNA sequencing experiment. (**c**) Flow cytometry staining of B cell phenotypes in NS, RBD-NP only stimulated, RBD-NP plus TLR7-NP stimulated, and TLR7-NP only stimulated organoid cultures from one representative of three donors on Day 8 (left) and the corresponding quantification of B cell differentiation towards pre-GC (CD38^+^CD27^-^), GC (CD38^+^CD27^+^), and plasmablast (CD38^+^CD27^++^) in all organoid cultures (right). (**d**) Flow cytometry staining of B cell phenotypes in NS, SARS-CoV-2 spike protein (spike) only stimulated, spike plus TLR7-NP stimulated, and spike plus TLR7-NP in the presence of ST2825 inhibitor stimulated organoid cultures from two donors on Day 9.

We then used pseudo-time trajectory analysis to better understand the machinery of plasmablast differentiation and antibody secretion stimulated by TLR7-NP. Starting from naive B cells, B-cell differentiation diverges into preGC/GC B or memory B cells, and both later converge into GC B cells, which eventually turn into plasmablasts (Extended Data Fig. 9a). TLR7-NP (red dot) enhanced the GC differentiation compared to other stimulations (grey and blue dots) (Extended Data Fig. 9b). We also observed a significant decrease in Pre-GCB, GC B and plasmablast subsets when the antigen was omitted (Extended Data Fig. 9c) or in the presence of a MyD88 inhibitor ST2825 (Extended Data Fig. 9d) in TOS, suggesting an essential role of MyD88 signalling in directly activating B-cell responses by TLR7-NP. Although the adjuvating effects from TLR7-NP in TOS were probably mediated by TLR signalling through MyD88, we cannot rule out a possible contribution of TLR-induced interleukin (IL)-1 family cytokine signalling that also uses MyD88 as an adaptor protein^42^.

## Minimized systemic toxicity

**Extended Data Fig. 10:**
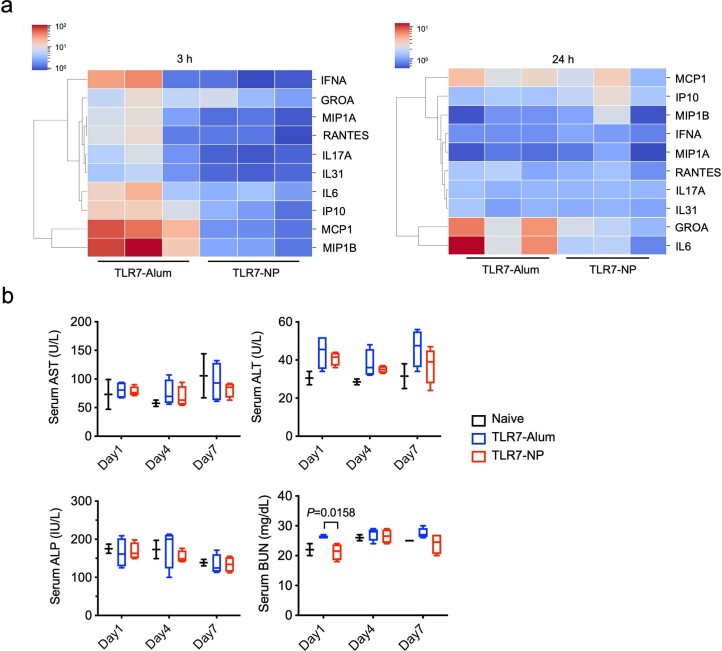
TLR7-NP adjuvant minimizes systemic toxicity. (**a**) The expression of top-10 elevated cytokines in the serum of mice (n = 3 mice per group) 3 hours and 24 hours after a single immunization of NP-OVA protein (50 μg) plus gardiquimod (20 μg) in either TLR7-Alum or TLR7-NP. Data are presented as log10 fold change of mean fluorescence intensity over naïve control mice. (**b**) The clinical chemistry of immunized mice at Day1, 4 and 7 post immunization (n = 2, 2, 2 mice for naïve, n = 4, 4, 4 mice for TLR7-Alum, n = 4, 4, 4 mice for TLR7-NP). Data are presented as Box and whiskers (Turkey), and analysed with two-sided, unpaired t test with Turkey’s multiple comparison.

We next evaluated the possibility of adjuvant-mediated toxicity by comparing TLR7-NP to TLR7–alum. TLR7–alum rapidly diffused into circulation within 3 h after a single injection, leading to acute inflammation, characterized by multiple elevated serum inflammatory cytokines compared with TLR7-NP (Extended Data Fig. 10a, left). In particular, GROA and IL-6 maintained a high serum level 24 h post-immunization (Extended Data Fig. 10a, right). We further evaluated the long-term toxicity by clinical chemistry at 1, 4, and 7 days post-immunization. The result showed a significantly increased blood urea nitrogen (BUN) enzyme level in the TLR7–alum-immunized group at day 1, indicating the acute kidney toxicities (Extended Data Fig. 10b), presumably caused by fast renal clearance of small molecular TLR7 agonist in this formulation. In contrast, TLR7-NP-adjuvanted vaccines showed negligible systemic inflammation and renal toxicity.

## Outlook

The immune response to vaccination is a function of a given vaccine’s spatiotemporal profile, in which the timing and localization of antigen and adjuvant control key features of vaccine-induced immunity^43^. Here we report a general strategy to enhance the breadth and magnitude of antibody responses elicited by vaccination, accomplished by tuning the adjuvants’ physicochemical properties to promote draining lymph node accumulation and produce a stronger and persistent stimulatory effect. This may also apply to other TLR7 agonist–adjuvants with stable particulate formulation, such as 3M-052/Alum and AS37. The TLR7-NP adjuvant presented in this study not only holds great potential for developing more potent vaccines against influenza strains or other viruses, but also provides a useful tool for understanding the complexities of immune regulation.

Immunodominance in B-cell responses results from a few dominant clones outcompeting other clones in the GCs to produce long-lived memory compartments^44^. The induction of antibody breadth is usually associated with overcoming immunodominance^45–47^. Although the factors affecting immunodominance in the GC B-cell response are not clear, previous studies have suggested that epitope accessibility, precursor frequency, antigen affinity, T-cell help and previous antigen exposure all influence antibody immunodominance^48–50^. As shown in our study here, the significantly increased ratio of Tfh/Tfr in mice immunized with TLR7-NP might enhance T-cell help to promote the survival of subdominant B-cell clones in GCs. In addition, TLR7-NP can target dLNs and enhance the uptake of gardiquimod by B cells to promote B-cell activation and extrafollicular plasmablast response. Therefore, TLR–NP might improve the recruitment of subdominant B cells to participate in GC response to promote GC clonal diversity, as suggested by Extended Data Fig. 7.

Although efforts toward vaccine development often focus on inducing effective antibody responses, it is well known that humoral and cellular immune responses synergize to enhance immune protection^27^. Notably, TLR7-NP generates potent CD8^+^ T-cell responses, which are not optimally induced by commonly used alum adjuvants in vaccines. This property could potentially accelerate the development of vaccines against pathogens that trigger substantial cellular immune responses, such as malaria, tuberculosis and leishmaniasis.

One considerable hurdle of vaccine adjuvant development is eliciting a sufficiently potent immune response while meeting the special safety standard requirement for administration to the general population. The use of nanoparticles enables the spatiotemporal delivery of small molecular gardiquimod to the dLNs to enhance immune responses while reducing the likelihood of adverse systemic effects. In general, we find that our TLR7-NP has multiple favourable characteristics for drug loading, size, biocompatibility and scalability, and thus seems well suited for clinical application.

## Methods

### Animals

Female C57BL/6J mice 8–12 weeks old were purchased from the Jackson Laboratory and used in all of the studies presented in this paper. All the animals were cared for in the Stanford Animal Facility under specific pathogen-free conditions, 12 h light/12 h dark cycle, temperatures of ~18–23 °C with 40–60% humidity. The study protocol was reviewed and approved by the University Administrative Panel on Laboratory Animal Care (APLAC, protocols 30883 and 32763). For all the mouse studies in this paper, mice were randomly distributed to different groups before treatment.

### Human samples

Whole tonsils from consented individuals undergoing surgery for obstructive sleep apnoea, hypertrophy or recurrent tonsillitis were collected in accordance with the Stanford University Institutional Review Board. Ethics approval was obtained from the Stanford University Institutional Review Board (protocols 30837 and 60741). Written informed consent was obtained from adult participants and from the legal guardians of children aged 0–17 years; written informed consent was also obtained from children aged 7 years and older. The participants did not receive compensation. Whole tonsils were collected after surgery, decontaminated and processed as needed for culturing^40^.

### Preparation and characterization of TLR7–PLA polymer conjugate

In a glovebox, gardiquimod (3.1 mg, 0.01 mmol) was dissolved in anhydrous THF (300 µl) and mixed with a THF solution (500 µl) containing (BDI-EI)ZnN(TMS)_2_ (BDI-EI is 2-((2,6-diethylphenyl) amido)-4-((2,6-diisopropylphenyl)-imino)-2-pentene) (6.5 mg, 0.01 mmol). The mixture was stirred for 15 min. Lactide (LA) (14.4 mg, 10 equiv.) was dissolved in THF (500 µl) and added to the stirred (BDI-EI)ZnN(TMS)_2_ mixture. The reaction proceeded in the glovebox overnight. After LA was completely consumed, the reaction was stopped by quenching the polymerization solution with cold methanol solution (30 µl). The polymer was precipitated with ether (10 ml), collected by centrifugation and dried by vacuum. The molecular weights of polymer conjugates are characterized by gel permeation chromatography experiments, determined from the dn/dc value assuming 100% mass recovery using ASTRA 7 software (v.7.1.3.15, Wyatt Technology).

### Whole-mouse and lymph node fluorescence imaging

Mice were subcutaneously (s.c.) immunized with alum-adsorbed OVA (50 μg) mixed with either AF647-labelled TLR7–alum or AF647-labelled TLR7-NP (equivalent gardiquimod dose, 20 nmol) in 100 µl PBS at tail base. Signals of AF647 were measured with a longitudinal whole-animal in vivo Lago spectral imaging system from day 0 to day 3. Images were collected on a Largo X imaging system and analysed with Aura imaging software (Spectral Instruments Imaging). Total radiance at the s.c. injection site was normalized to the initial fluorescence signal at day 0 taken 30 min after injection. Inguinal dLNs were excised at various time points and whole-tissue fluorescence was measured by a Lago spectral imaging system at an excitation wavelength of 640 nm and an emission wavelength of 690 nm. Values represent the integrated fluorescence intensity.

### Cellular uptake study

Mice were s.c. immunized with alum-adsorbed OVA (some are labelled with AF488 as noted, 50 μg) mixed with either AF647-labelled TLR7–alum or AF647-labelled TLR7-NP (equivalent gardiquimod dose, 20 nmol) in 100 µl PBS at tail base. Inguinal dLNs were collected and gently dissociated with a 3-ml syringe plunger thumb rest and digested with 1 mg ml^−1^ collagenase type 4 for 20–25 min at 37 °C. Reactions were stopped with 2 mM EDTA and single-cell suspensions were prepared by passing through 40 µm cell strainers. The cells were stained with Ghost Dye Violet 510 (Tonbo Biosciences), and then washed, blocked with Fc-blocker (clone 2.4G2, BD Bioscience) before staining with markers, including CD8 (clone 53.67, BD Biosciences), PDCA1 (clone 927, BD Biosciences), Ly6C (clone HK1.4, Biolegend), CD11b (clone M1/70, Biolegend), CD138 (clone 281-2, BD Biosciences), CD11c (clone N418, Biolegend), MHCII (clone M5/114.15.2, Biolegend), Ly6G (clone 1A8, Biolegend), F4/80 (clone BM8, Biolegend), CD40 (clone 3/23, Biolegend), SiglecF (clone E50-2440, BD Biosciences), CD103 (clone 2-E7, eBioscience), CD19 (clone 1D3, Biolegend) and CD86 (clone P03, Biolegend), and then fixed with 1.5% PFA, and collected using BD FACS Diva v.8.01 software associated with a BD LSRII flow cytometer. Data were analysed with FlowJo 10 software. The gating strategy for cells of myeloid lineage is shown in Extended Data Fig. 3. B cells were gated on live, single CD19^−^ CD3^+^ cells.

### Phenotypic analysis of B cells and follicular T cells

Mice were s.c. immunized with alum-adsorbed NP–OVA (50 µg) and TLR7–alum or TLR7-NP (equivalent gardiquimod dose, 20 µg) in 100 µl PBS at tail base. Inguinal dLNs were excised at day 4, day 7, day 14 and day 22 to prepare single-cell suspensions. For flow cytometry analysis of GC B cells, follicular T cells (TFH) and plasmablasts, cells from the dLNs were stained with Ghost Dye Violet 510 (Tonbo Biosciences). Cells were then washed and blocked with Fc-blocker (clone 2.4G2, BD Bioscience) prior to staining with markers, including CD19 (clone 1D3/CD19, Biolegend), CD38 (clone 90, BD Biosciences), CD95 (clone Jo2, BD Biosciences), CD138 (clone 281-2, BD Biosciences), CD44 (clone IM7, BioLegend), CD3 (clone 17A2, BioLegend), CD4 (clone GK1.5, BioLegend), CXCR5 (clone L138D7, BioLegend) and PD1 (clone 29F.1A12, BioLegend). After staining, cells were washed and fixed with 1.5% PFA. Stained cells were collected using BD FACS Diva v8.01 software associated with a BD LSRII flow cytometer. Data were analysed with FlowJo 10 software. The gating strategy consisted of gating GCBC on live single CD3^−^CD19^+^CD95^+^CD38^−^ cells, TFH on live single CD19^−^CD3^+^CD4^+^CXCR5^+^PD1^hi^, and plasmablasts on live single CD138^+^CD44^+^ cells.

### Phenotypic and functional analysis of T cells

For analysis of T cells, cells from dLNs were collected and prepared as single-cell suspensions. The cells were washed, blocked with Fc-blocker (clone 2.4G2, BD Bioscience) on ice for 10 min, and stained with PE-labelled H2-K^b^-SIINFEKL tetramer (MBL International) at room temperature for 1 h. Cells were then washed twice and blocked with Fc-blocker (clone 2.4G2, BD Bioscience), stained with LIVE/DEAD Fixable Aqua Dead Cell Stain and surface markers, including CD45 (clone 30-F11, BioLegend), TCRβ chain (clone H57-597, Biolegend), CD3 (clone 17A2, BioLegend), CD8α (clone 53-6.7, BD Biosciences), CD4 (clone GK1.5, BD Biosciences), CD44 (clone IM7, BioLegend), CD62L (clone MFL^−^14, BioLegend) and CD69 (clone H1.2F3, BioLegend), and analysed on a BD LSRII flow cytometer. For functional analysis, cells from dLNs were plated in 24-well plates in complete T-cell media with eBioscience Cell Stimulation Cocktail (plus protein transport inhibitors) for 5 h. After stimulation, the cells were collected, washed, blocked with Fc-blocker and then stained with LIVE/DEAD Fixable Aqua Dead Cell Stain and surface markers, including CD45 (clone 30-F11, BioLegend), CD3 (clone 17A2, BioLegend), TCRβ chain (clone H57-597, Biolegend), CD4 (clone GK1.5, BD Biosciences), CD8α (clone 53-6.7, BD Biosciences) and CD279 (PD-1) (clone 29F.1A12, BioLegend), and then fixed using eBioscience Foxp3/Transcription Factor Staining Buffer Set according to the manufacturer’s instructions. Cells were then washed, permeabilized, stained for function markers, including Foxp3 (clone MF-14, Biolegend), IFN-γ (clone XMG 1.2, Biolegend), Granzyme B (clone QA16A02, Biolegend) and BCL6 (clone K112-91, BD Biosciences), and collected using BD FACS Diva v.8.01 software associated with a BD LSRII flow cytometer. Data were analysed with FlowJo 10 software. For the gating strategy, Tem SIINFEKL^+^ CD8 T cells were gated on live single CD3^+^TCRβ^+^CD4^−^CD8^+^ SIINFEKL^+^CD44^+^CD62L^−^ cells, CD69^hi^ SIINFEKL^+^ CD8 T cells were gated on CD3^+^TCRβ^+^CD4^−^CD8^+^ SIINFEKL^+^CD69^+^ cells, and Gzmb^+^CD8 T cells were gated on CD3^+^TCRβ^+^CD4^−^CD8^+^ Gzmb^+^ cells. Regulatory follicular T cells (Tfr) were gated on CD3^+^ CD4^+^ CD8^−^BCL6^+^PD1^hi^ FoxP3^+^ cells.

### Mouse lung tissue isolation and processing

Lung isolation was performed as described^51,52^ with some modifications. Briefly, killed mice were perfused via the right cardiac ventricle with PBS. Harvested lungs were dissected into gentleMACS C tubes (Miltenyi) containing 4 ml of a mixture of collagenase (25 μg ml^−1^ liberase^M^, Roche) and DNaseI (10 μg ml^−1^, Sigma) in PBS containing 2% FBS. Then lungs were dissociated with a preset program m_lung_02_01 using a gentleMACS octo dissociator (Miltenyi) and incubated for 30 min at 37 °C. Then they were homogenized with the gentleMACS program m_lung_02_01 in the presence of 10 mM EDTA. Single-cell suspensions were prepared with 100 μm cell strainers. Red blood cells were lysed with Ack lysing buffer (Gibco). Cells were resuspended in 4 ml 36% Percoll (GE Healthcare) and washed once for further experiments. To further distinguish T cells in the lung vasculature versus the lung parenchyma, mice were injected with 3 µg APC-conjugated anti-mouse CD8α antibody (clone 53-6.7, Biolegend) through tail vein injection 3 min before lung perfusion and dissection. The single-cell suspension was stimulated, fixed and stained with antibodies for anti-CD16/CD32 (clone 2.4G2, BD Biosciences), CD45 (clone 30-F11, BioLegend), TCRβ (clone H57-597, Biolegend), CD3 (clone 17A2, BioLegend), CD8α (BUV805, clone 53-6.7, BD Biosciences), CD4 (clone GK1.5, BD Biosciences), CD44 (clone IM7, BioLegend), CD62L (clone MFL-14, BioLegend) and Granzyme B (clone QA16A02, Biolegend). Data were collected using BD FACS Diva v.8.01 software associated with a BD LSRII flow cytometer. Gzmb^+^CD8 T cells in the lungs were gated on single live CD45^+^CD3^+^TCRβ^+^CD4^−^CD8^+^ Gzmb^+^ cells and Gzmb^+^ lung tissue-resident CD8 T cells were gated on CD45^+^CD3^+^ TCRβ^+^CD8(APC)^−^CD8(BUV805)^+^ Gzmb^+^ cells.

### Immunofluorescence

Cryostat sections (7 μm) made from OCT (TissueTek) embedded dLNs were fixed with 2% PFA for 20 min, and then washed and blocked in blocking buffer (PBS with 1% BSA, 0.3% Triton-100, Fc-blocker and 5% rat serum and mouse serum). Sections were then stained in blocking buffer with biotin-labelled anti-mouse IgD (clone 11-26c, eBioscience) and PE-labelled anti-mouse BCL6 antibodies (clone K112-91, BD Biosciences), and subsequently stained with AF488-conjugated streptavidin (Thermo Fisher Scientific). Fluorescent images were captured using a 4× objective on a fluorescence microscope (Keyence).

For imaging the distribution of TLR7-NP in the dLNs, AF647-labelled TLR7-NP was s.c. injected into mice (*n* = 2) at the tail base. dLNs were harvested 48 h later. Tissue sections were stained with anti-mouse IgD_Al488 (clone 11-26c, SouthernBiotech), CD4_BV421 (clone GK1.5, BioLegend) and CD35_biotin (clone 8C12, BD Biosciences). Streptavidin_A555 (Invitrogen, catalogue number S32355, 1:100) was used to detect biotin. Images were captured using a 20× objective on a fluorescence microscope (Leica, DMi8).

### ELISA analysis of antibody titres

Mice were s.c. immunized with alum-adsorbed recombinant HA (10 µg) or recombinant full-length SARS-CoV-2 spike protein (5 µg) with TLR7–alum or TLR7-NP (equivalent gardiquimod dose, 20 µg) in 100 µl PBS at tail base at week 0 and boosted at week 3. Sera were collected by face bleeding 2 weeks following either priming or boosting for enzyme-linked immunosorbent assay (ELISA) assays to measure anti-HA titres or anti-SARS-CoV-2 titres. High protein binding plates (Costar) were directly coated with the antigen of interest (2 µg ml^−1^, 50 µl) overnight. The plates were blocked with the blocking buffer for 1 h, washed and cultured with diluted mouse sera (1:500 or 1:2,500) for 2 h. Horseradish peroxidase (HRP)-conjugated anti-mouse secondary antibodies to IgG (SouthernBiotech, 1031-05, 1:4,000) was used to detect bound antibodies. Plates were developed with TMB substrate solution (Thermo Scientific), quenched with sulfuric acid and read at 450 nm with a microplate reader (Bio-Rad). In all ELISA assays, unvaccinated mouse serum was used as negative control.

### Influenza viral challenge

Mice were s.c. immunized with alum-adsorbed H1 (NC99, 10 µg) mixed with TLR7–alum or TLR7-NP (equivalent gardiquimod dose, 20 µg) in 100 µl PBS at tail base. At week 7, the immunized mice were anaesthetized and intratracheally infected with the heterologous influenza A/PR/8/34 H1N1 (Charles River, catalogue number 10100374, lot number 4XP201023) in 20 μl sterile PBS (1/40,000 dilution from 10^7^ p.f.u. per ml virus stock). Virus titres in the stock were determined with plaque assay; detailed methods are described in the Supplementary Information. Body weight and survival of mice were monitored for 12 days after the challenge. The endpoint was defined as a body weight drop >25.5% or natural death. The body weight drop curve stopped when *n* > 2 mice died in the group.

### ELISPOT analysis

Bone marrow ELISPOTs were performed at week 52 after immunizations according to the manufacturer’s instructions (MabTech). The plates were generally coated with antigens (5 µg ml^−1^, 50–100 µl) overnight at 4 °C. Then the plates were washed and blocked for the ELISPOT assays. Single cells were isolated from bone marrow of both upper and lower leg bones and further incubated with prepared antigen-coated plates at 37 °C for 18 h. After washing the plates, an HRP-conjugated anti-mouse IgG antibody (SouthernBiotech, 1031-05, 1:2,500) was added, and antigen-specific responses were detected by the detection kit (BD Biosciences, number 551951).

### Human tonsil organoid stimulation

The tonsil cells were cryopreserved as previously described^40^. Cryopreserved cells from seven paediatric donors (including both male and female, aged from 5 to 10 years old) and one adult donor (male, 68 years old) were thawed and cultured. For culture of cryopreserved cells, aliquots were thawed into complete medium, enumerated and resuspended to 6 × 10^6^ cells per ml. Cells were plated, 100 μl per well, into permeable (0.4 μm pore size) membranes (24-well size PTFE or polycarbonate membranes in standard 12-well plates with single-well receiver trays; Corning or Millipore), with the lower chamber consisting of complete medium (1 ml) supplemented with 0.5 μg ml^−1^ of recombinant human B-cell-activating factor (BAFF; BioLegend) every 3 or 4 days. Antigen including either full-length SARS-CoV-2 spike protein (2.5 μg per well) or RBD-NP (2.0 μg per well) with or without adjuvant TLR7-NPs (5 μg per well) was added directly to the cell-containing portion of the culture set-up. Some experiments also involved the ST2825 inhibitor. Cultures were incubated at 37 °C, 5% CO_2_ with humidity. Cells from organoids were harvested after 14 day culture, stained with Human TruStain FcX (Biolegend), LIVE/DEAD Fixable Aqua Dead Cell Stain and surface markers, including CD45 (clone HI30, Biolegend), CD3 (clone HIT3a, Biolegend), CD19 (clone 4G7, Biolegend), CD8 (clone SK1, BD Biosciences), CD4 (clone RPA-T4, BioLegend), CD38 (clone HB-7 Biolegend) and CD27 (clone LG.3A10, Biolegend), and analysed by flow cytometry. Gating strategy: preGC B cells (CD45^+^CD19^+^CD3^−^CD38^+^CD27^−^), GC B cells (CD45^+^CD19^+^CD3^−^CD38^+^CD27^mid^), plasmablasts (CD45^+^CD19^+^CD3^−^CD38^+^CD27^hi^). Supernatants from the lower chamber were harvested for measuring SARS-CoV-2 spike-specific antibodies by ELISA. HRP-conjugated anti-human secondary antibodies to IgA and IgM (SouthernBiotech, IgA catalogue number 2050-05, IgM catalogue number 2020-05) were used to detect bound antibodies.

### BD Rhapsody single-cell targeted RNA sequencing and data analysis

Tonsil organoid cells from one donor (a 6-year-old female) cultured for different numbers of days and with different stimulants were FACS-sorted, library prepared and sequenced in one batch. They were first stained with oligonucleotide-conjugated Sample Tags (BD Human Single-Cell Multiplexing Kit, catalogue number 633781) and LIVE/DEAD Aqua Zombie stain, and with surface markers following the manufacturers’ protocol. Live B cells (CD45^+^CD19^+^CD3^−^) were FACS-sorted from the barcoded samples and pooled. Barcoded samples were then washed and spun down at 350*g* for 10 min and pooled. The pooled sample was then stained concurrently with a panel of 32 oligonucleotide-conjugated antibodies for the AbSeq Stain in BD stain buffer for 30 min on ice. Samples were then spun down at 350*g* for 10 min and washed three times. The pellet was resuspended in Rhapsody Sample Buffer for cell capture. Cell capture and library preparation were completed using the BD Rhapsody Targeted mRNA and AbSeq Reagent Kit (catalogue number 633774). Briefly, cells were captured with beads in a microwell plate, followed by cell lysis, bead retrieval, cDNA synthesis, template switching and Klenow extension, and library preparation in the Stanford Human Immune Monitoring Centre following the BD Rhapsody protocol. Libraries were prepared for sample tags, AbSeq and targeted mRNA using the customized Immune Response panel. Sequencing was completed on a NovaSeq (Illumina) at Novogene USA.

The Rhapsody raw data were first preprocessed by the Seven Bridges Genomics online platform using the BD Rhapsody Targeted Analysis Pipeline to align genes and calculate molecular counts with molecular index correction. After the preprocessing, the single-cell Rhapsody count tables, which were composed of 493 genes and 32 surface markers from the different time points, and the stimulants were imported in Seurat package of R (v.4.2.1)^53^ for the following processing. Both the gene and abseq counts were log_1_*p* transformed. Principal component analysis with 30 dimensions was used to dimensionally reduce the gene and abseq combined count data. Graphically based clustering and manual annotation were applied to identify B-cell subsets. To identify the genes that are significantly differentially expressed between the two treatments, we used Wilcoxon rank-sum tests^54^ with the Benjamini–Hochberg adjusted *P*-values below 0.05 and absolute values of the log_2_ of the fold change between the average expression of the raw molecular counts above 0.25. The ‘biological processes’ GO terms of the significantly differentially expressed genes were calculated using DAVID^55,56^ and the GO terms were shown with a false discovery rate of <0.05. To estimate the B-cell differentiation lineage, we ran the pseudotime trajectory analysis using tSpace in R^57^. tSpace used the top 50 principal components of the dataset we obtained earlier as the input. The output is a multidimensional trajectory space in which each cell–cell distance represents their lineage pseudotime distance. This pseudotime trajectory cell space is then visualized using the dimensionality reduction visualization UMAP plot to best present the B-cell differentiation lineage.

### Bulk BCR sequencing and diversity calculation

Mice (*n* = 3 per group) were immunized with TLR7–alum- or TLR7-NP-adjuvanted recombinant HA (H1 HA PR8) as described above. GC B cells were sorted from draining lymph nodes of immunized mice 14 days post-immunization and DNA was extracted using a QIAmp DNA Micro Kit (Qiagen). Sequencing of mouse IgH chains was performed using the immunoSEQ Assay (Adaptive Biotechnologies). The diversity index iChao1 is calculated using the Adaptive Biotechnologies Immuoseq Analyzer software. iChao1 is a non-parametric estimator of the lower bound of the total number of unique templates within an individual’s repertoire. The lower bound is the minimum number of unique templates predicted to be within an individual’s repertoire, with a 95% confidence interval^58–60^.

### Statistics and reproducibility

Statistics were analysed using GraphPad Prism9 software. Detailed statistical analysis methods are included in the figure legends. No statistical methods were used to predetermine sample sizes, but our sample sizes are similar to those reported in previous publications. Data distribution was assumed to be normal, but this was not formally tested. Data are either combined from multiple individual experiments or were from one representative of at least two individual experiments. One mouse in the SARS-CoV-2 immunization study was euthanized by staff due to skin lesions, and was therefore excluded from analyses. The investigators were not blinded to allocation during experiments and outcome assessment.

### Reporting summary

Further information on research design is available in the Nature Portfolio Reporting Summary linked to this article.

## Online content

Any methods, additional references, Nature Portfolio reporting summaries, source data, extended data, supplementary information, acknowledgements, peer review information; details of author contributions and competing interests; and statements of data and code availability are available at 10.1038/s41563-022-01464-2.

## Supplementary information

Supplementary Information Supplementary Information

Reporting Summary

## Data Availability

Data supporting the findings of this study are available in the article and its supplementary files. Single-cell RNA sequencing data are deposited in NCBI’s Gene Expression Omnibus and are accessible through GEO Series accession number GSE217918. Bulk BCR sequencing data are deposited in https://clients.adaptivebiotech.com/immuneaccess with 10.21417/QY2022NM. Source data are provided with this paper.

## Source data

Source Data Fig. 1 Statistical source data for Fig. 1b–d.
Source Data Fig. 2 Statistical source data for Fig. 2a–f.
Source Data Fig. 3 Statistical source data for Fig. 3a–g.
Source Data Fig. 4 Statistical source data for Fig. 4b–d,f,g.
Source Data Fig. 5 Statistical source data for Fig. 5b–d.
Source Data Fig. 6 Statistical source data for Fig. 6b,c.
Source Data Extended Data Figs. 1 and 4–10 Statistical source data for Extended Data Figs. 1a, 4b, 5a, 6a,b, 7, 8a,b, 9c and 10a,b.

## Code availability

The custom code for analysing tonsil single-cell RNA-seq data is available at https://github.com/ttsunmeng/scRNA-seq-codes/blob/main/tonsil_organoid_Bcell_IMD090_RBDNP_TLR7NP.R.

The standard code for plotting serum cytokine with seaborn.heatmap of python is available upon request.
