# Supplementary material for: A TLR7-nanoparticle adjuvant promotes a broad immune response against heterologous strains of influenza and SARS-CoV-2
Source: Nat Mater. 2023 Jan 30;22(3):380–90. doi: 10.1038/s41563-022-01464-2 (PMC9981462; doi:10.1038/s41563-022-01464-2)
Supplement: Supplementary file 1 — Supplementary Information [file 41563_2022_1464_MOESM1_ESM.pdf]

nature materials

Article

https://doi.org/10.1038/s41563-022-01464-2

# A TLR7-nanoparticle adjuvant promotes a broad immune response against heterologous strains of influenza and SARS-CoV-2

In the format provided by the authors and unedited

**Table of Contents**

Reagents
Instrument
Supplementary Methods
Reference

## Reagents

L-Lactide (LLA) was purchased from TCI America (Portland, OR, USA). It was recrystallized three times in anhydrous toluene and stored at -30 $^{o}$C in glove box. mPEG-PLGA (LG 50:50 (w:w), Mw 5000:10000Da) was purchased from Akina, Inc. (West Lafayette, IN, USA). TLR7 agonist (gardiquimod) was purchased from Sigma-Aldrich or InvivoGen (San Diego, CA, USA). Alexa Fluor™ 647 NHS Ester (Succinimidyl Ester) was purchased from Thermofisher (Waltham, MA, USA). ((BDI)Zn catalysts and precursor were synthesized according to the previous report using similar procedure[1, 2]. All other chemical reagents were purchased from Sigma-Aldrich and used as received except where otherwise noted. Recombinant SARS-CoV-2 protein (Spike, S1, RBD) and variants were purchased from Sino Biological, Inc (Beijing, China). H2-K$^{b}$ SIINFEKL tetramer-PE was purchased from MBL international corporation (Woburn, MA, USA).

## Instrument

Gel permeation chromatography (GPC) experiments were performed on a system equipped with an isocratic pump (Model 1260, Agilent Technology, Santa Clara, CA, USA), a DAWN HELEOS multiangle laser light scattering (MALLS) detector, and an Optilab rEX refractive index detector (Wyatt Technology, Santa Barbara, CA, USA). The detection wavelength of HELEOS was set at 658 nm. Separations were performed by serially connected size exclusion columns (three PLgel MIXED-B columns, 10 μm, 7.5 × 300 mm, Agilent, Santa Clara, CA, USA) which were maintained at a temperature of 40 ºC using DMF containing 0.1 M LiBr as the mobile phase at a flow rate of 0.7 mL/min. The MALS detector was calibrated using pure toluene and then was used for the determination of the absolute molecular weights (MWs). All sample solutions were filtered by a 0.45 μm PTFE filter before the injection. The molecular weights of polymers were determined from the dn/dc value assuming 100% mass recovery using ASTRA 7 software (Version 7.1.3.15, Wyatt Technology). MALDI-TOF spectra were taken on a Bruker Ultra Flextreme equipped with a 337 nm nitrogen laser. The sizes and the size distributions of the NPs were determined on a ZetaPALS dynamic light scattering (DLS) detector (15 mW laser, incident beam = 676 nm, Brookhaven Instruments, Holtsville, NY, USA). Flow data were acquired on LSRII or LSRII.2 flow cytometer (BD biosciences). Cells were sorted on FACSAria Fusion (BD Biosciences). The absorbance of ELISA assay was measured

on Microplate Reader (Bio-Rad). The whole animal in vivo imaging was performed on Spectral Instruments Imaging (Tuscon, AZ, USA).

**Supplementary Methods**

**Preparation and characterization of TLR7-NP**

Particle preparation: A 100-µL DMF mixture of TLR7–PLA (50 µL, 10 mg/mL) and PEG-PLGA (50 µL, 10 mg/mL) added dropwise to rapidly stirred nanopure water (2 mL). The resulting TLR7- NP was washed with DI water and collected by ultrafiltration (15 min, 4,000 × g, Ultracel membrane with 10,000 NMWL; Millipore.

Particle size measurement: The sizes and the size distributions of TLR7-NP was determined on a ZetaPALS dynamic light scattering (DLS) detector (Brookhaven Instruments).

Release kinetics measurement: TLR7-NPs were resuspended in FBS/PBS buffer (0.5 mL, 1:1, v/v) in a dialysis tube with a cut of 1kD and immersed in a tube containing PBS (4.5 mL). Sample (0.5 mL) was collected from the tube at each time point while supplementing with 0.5 mL PBS at the same time at 0, 1, 2, 3, 4, 5 days. The gardiquimod concentration was measured by UV spectrometer at $\lambda$ = 321nm. The 100 % release control was achieved by co-incubating the above NP with 0.1 M NaOH at 37$^{o}$ C for 6 h and measured with Cary 60 UV-Vis spectrometer (Agilent Technologies).

**Preparation of AF647-labeled TLR7-Alum:** AF647-NHS (0.5 mg, 0.4 µmol) was dissolved in dd$H_2O$ (10 µL) and then mixed with gardiquimod (132 µg, 0.42 µmol) in dd$H_2O$ (10 µL), stirred overnight. The reaction was purified by flashing on a C18 column (Teledyne ISCO, 30 g, Lincoln, NE, USA) with $NH_4HCO_3$ (5mM in dd$H_2O$) and acetonitrile (25% ~ 80%, v/v) and then lyophilized to get the AF647-gardiquimod. AF647-labeled TLR7-Alum is prepared by mixing AF647-gardiquimod with Alhydrogel®, following the same procedure for preparing TLR7-Alum.

**Preparation of AF647-labeled TLR7-NP:** AF647-NHS (0.5 mg, 0.4 µmol) was dissolved in dd$H_2O$ (10 µL) and then mixed with TLR7-PLA (2.0 mg, 0.5 µmol, 1.2 equiv) in DMF (40 µL), stirred overnight. The reaction was purified by dialysis with a dialysis kit with a cut of 3.5 kD and lyophilized to get the AF647-labeled TLR7-PLA polymer. Then AF647-TLR7-NP was prepared following the same procedures for preparation TLR7-NP.

**Neutralization assay**

Mouse sera were treated with receptor destroying enzyme (RDE) from Vibrio cholerae (Sigma, # N7885) overnight at 37 °C and then heat-treated for 30 min at 56 °C. Serum was diluted in virus dilution medium (1:200, DMEM with 1 ug/ml TPCK treated trypsin, 2% BSA) and incubated with PR8 (2 X 104 PFU) for 1 h in 37 °C. The mixture of serum and virus was added on top of MDCK cells in 24-well plates for 1 h in 37 °C. After 1 X PBS wash, 1ml virus dilution medium was added into each well, and cells were kept for 18 h in 37 °C. The RNA in the cells was analyzed by Taqman-based RT-PCR with PR8 specific primers

(flu-F: AGACCRATCYTGTCACCTCTGAC, flu-R: AGGGCATTYTGRACAAAICGTCTACG, flu-P: FAM-TCACCGTGCCCAGTGAGCGAGGACTGC-BHQ-1).

The RNA purified from virus stock served as the standard.

**Immune toxicity evaluation**

Mice were s.c. immunized with Alum-adsorbed NP-OVA (50 µg) and TLR7-Alum or TLR7-NP (equivalent gardiquimod dose: 20 µg) in 100µL PBS at tail base at Day 0. Naïve mice were as control. Serum (n=3 mice per group) was collected 3 h and 24 h post immunization. Cytokines and chemokines in the serum were measured using a Luminex bead-based ELISA following the manufacturer's instructions. Blood (n=2 mice for naïve, N=4 mice for TLR7-Alum or TLR7-NP) was collected at Day 1, 4 and 7 for clinical chemistry measurement. Data was plotted by seaborn. heatmap of python (V2.7).

**Reference**

1. Yin, Q. *et al.* Pamidronate functionalized nanoconjugates for targeted therapy of focal skeletal malignant osteolysis. *Proc Natl Acad Sci U S A* **113**, E4601-4609 (2016).

2. Tong, R. & Cheng, J. Ring-opening polymerization-mediated controlled formulation of polylactide-drug nanoparticles. *J Am Chem Soc* **131**, 4744-4754 (2009).
