# Supplementary material for: A TLR7-nanoparticle adjuvant promotes a broad immune response against heterologous strains of influenza and SARS-CoV-2
Source: Nat Mater. 2023 Jan 30;22(3):380–90. doi: 10.1038/s41563-022-01464-2 (PMC9981462; doi:10.1038/s41563-022-01464-2)
Supplement: Supplementary file 2 — Reporting Summary [file 41563_2022_1464_MOESM2_ESM.pdf]

nature portfolio

Corresponding author(s): Mark M. Davis, Bali Pulendran

Last updated by author(s): 11-17-2022

Reporting Summary

Nature Portfolio wishes to improve the reproducibility of the work that we publish. This form provides structure for consistency and transparency in reporting. For further information on Nature Portfolio policies, see our Editorial Policies and the Editorial Policy Checklist.

Statistics

For all statistical analyses, confirm that the following items are present in the figure legend, table legend, main text, or Methods section.

n/a	Confirmed	
☐	☒	The exact sample size (*n*) for each experimental group/condition, given as a discrete number and unit of measurement
☐	☒	A statement on whether measurements were taken from distinct samples or whether the same sample was measured repeatedly
☐	☒	The statistical test(s) used AND whether they are one- or two-sided<br>*Only common tests should be described solely by name; describe more complex techniques in the Methods section.*
☒	☐	A description of all covariates tested
☐	☒	A description of any assumptions or corrections, such as tests of normality and adjustment for multiple comparisons
☐	☒	A full description of the statistical parameters including central tendency (e.g. means) or other basic estimates (e.g. regression coefficient) AND variation (e.g. standard deviation) or associated estimates of uncertainty (e.g. confidence intervals)
☐	☒	For null hypothesis testing, the test statistic (e.g. *F*, *t*, *r*) with confidence intervals, effect sizes, degrees of freedom and *P* value noted<br>*Give P values as exact values whenever suitable.*
☒	☐	For Bayesian analysis, information on the choice of priors and Markov chain Monte Carlo settings
☒	☐	For hierarchical and complex designs, identification of the appropriate level for tests and full reporting of outcomes
☒	☐	Estimates of effect sizes (e.g. Cohen's *d*, Pearson's *r*), indicating how they were calculated

Our web collection on statistics for biologists contains articles on many of the points above.

Software and code

Policy information about availability of computer code

Data collection	Flow cytometry data were collected using BD FACS Diva V.8.01 software associated with BD LSRII flow cytometer. ELISA plates were measured by Bio-Rad Microplate Reader. Fluorescence measurements in mice and lymph nodes were collected on a Largo X imaging system.
Data analysis	Flowjo v10 was used for flow cytometry analysis. GraphPad Prism V9 was used for data analysis and plots. Serum cytokine heatmap was plotted by seaborn. heatmap of python (V2.7). Fluorescence images were analyzed with Aura imaging software. Polymer molecular weight was analyzed using ASTRA 7 software (Version 7.1.3.15). For scRNA-seq, the raw data was first preprocessed by the Seven Bridges Genomics online platform and then analyzed in Seurat package of R (V4.2.1). For Bulk-seq of BCR, the data was calculated using the Adaptive Biotechnologies Immuoseq Analyzer Software.

For manuscripts utilizing custom algorithms or software that are central to the research but not yet described in published literature, software must be made available to editors and reviewers. We strongly encourage code deposition in a community repository (e.g. GitHub). See the Nature Portfolio guidelines for submitting code & software for further information.

Data

Policy information about availability of data

All manuscripts must include a data availability statement. This statement should provide the following information, where applicable:

- Accession codes, unique identifiers, or web links for publicly available datasets
- A description of any restrictions on data availability
- For clinical datasets or third party data, please ensure that the statement adheres to our policy

Data supporting the findings of this study are available in the source data files.
Single-cell RNA sequencing data are deposited in NCBI's Gene Expression Omnibus (Edgar et al., 2002) and are accessible through GEO Series accession number

GSE217918. (https://www.ncbi.nlm.nih.gov/geo/query/acc.cgi?acc=GSE217918).
Bulk BCR sequencing data are deposited in https://clients.adaptivebiotech.com/immuneaccess with DOI: 10.21417/QY2022NM.

Field-specific reporting

Please select the one below that is the best fit for your research. If you are not sure, read the appropriate sections before making your selection.

☒ Life sciences ☐ Behavioural & social sciences ☐ Ecological, evolutionary & environmental sciences

For a reference copy of the document with all sections, see nature.com/documents/nr-reporting-summary-flat.pdf

Life sciences study design

All studies must disclose on these points even when the disclosure is negative.

Sample size	Sample sizes for animal studies were based on the prior work (Ref: Irvine, Nat Med, 2020) without using additional statistical estimations. Sample sizes for experiments relying on limited human clinical samples were determined upon availability.
Data exclusions	One mouse in the SARS-COV-2 Immunization study was euthanized between week 2 and week 5 due to skin lesion caused by fighting.
Replication	All murine experiments report pooled results from multiple experiments or data shown is one representative of at least two experiments. All attempts at replication were successful.<br>Different human donors were used to show the inter-variation in the ability to respond to the vaccine tested.
Randomization	Animals were randomly distributed to different groups before treatment. To evaluate the SARS-CoV-2 vaccine efficacy in human tonsil organoids, we chose the human samples naive to the tested vaccine.
Blinding	During the experiments, investigators need to know the treatment for each group especially when the experiments were performed by the same investigator. All data were acquired and analyzed by softwares with objective standard. So blinding is not relevant to the data analysis.

Reporting for specific materials, systems and methods

We require information from authors about some types of materials, experimental systems and methods used in many studies. Here, indicate whether each material, system or method listed is relevant to your study. If you are not sure if a list item applies to your research, read the appropriate section before selecting a response.

Materials & experimental systems

n/a	Involved in the study	
☐	☒	Antibodies
☒	☐	Eukaryotic cell lines
☒	☐	Palaeontology and archaeology
☐	☒	Animals and other organisms
☐	☒	Human research participants
☒	☐	Clinical data
☒	☐	Dual use research of concern

Methods

n/a	Involved in the study	
☒	☐	ChIP-seq
☐	☒	Flow cytometry
☒	☐	MRI-based neuroimaging

Antibodies

Antibodies used	The following antibodies were used in the mouse study:<br>anti-CD16/CD32 (BD bioscience, Cat#: 553141, clone 2.4G2,1:100), CD8α(clone 53.67, BD Bioscience,Cat#612898, 1:200), PDCA1 (clone 927, BD Bioscience, Cat#747602, 1:200), Ly6C (clone HK1.4, Biolegend, Cat#128041, 1:500), CD11b (clone M1/70, Biolegend, Cat# 101259, 1:500), CD138 (clone 281-2, BD Bioscience, Cat#563147, 1:100), Ghost Dye™ Violet 510 (Tonbo, Tonbo Biosciences, 1:300), CD11c (clone N418, Biolegend, Cat# 117343, 1:400), MHCII (Clone M5/114.15.2, Biolegend, Cat#107622, 1:400), Ly6G (Clone 1A8, Biolegend, Cat#127624, 1:400), F4/80 (clone BM8, Biolegend, Cat#123116, 1:200), CD40 (Clone 3/23, Biolegend, Cat#124622, 1:400), SiglecF (clone E50-2440, BD Bioscience, cat#562757, 1:400), CD103 (clone 2-E7, eBioscience, cat# 12-1031-82, 1:200), CD19 (clone 1D3, Biolegend, cat# 152406, 1:200), CD86 (Clone P03, Biolegend, cat#105110, 1:400), CD19 (clone 1D3/CD19, Biolegend, Cat# 152406, 1:200), CD38 (clone 90, BD Biosciences, cat#740245, 1:200), CD95 (clone Jo2, BD Biosciences, Cat#: 557653, 1:200), CD138 (clone 281-2, BD Biosciences, Cat#: 563147, 1:100), CD44 (clone IM7, BioLegend, cat#103028, 1:300), CD3 (clone 17A2, BioLegend, cat# 100216, 1:100), CD4 (clone GK1.5, BioLegend, cat# 100469, 1:200), CXCR5 (clone L138D7, BioLegend, cat #145529, 1:100), PD1 (clone 29F.1A12, BioLegend, cat# 135228, 1:100), CD45 (clone 30-F11, BioLegend, cat#: 103140, 1:100), TCRβ chain (clone H57-597, Biolegend, cat#: 109240, 1:100), CD3 (clone 17A2, BioLegend, cat#: 100216, 1:100), CD8α (clone 53-6.7, BD Biosciences, cat#: 564920, 1:100), CD4 (clone GK1.5, BD Biosciences, cat#: 565974, 1:100), CD44 (clone IM7, BioLegend, cat#: 103028, 1:100), CD62L (clone MFL-14, BioLegend, cat#: 104438, 1:100), CD69 (clone H1.2F3, BioLegend, cat#: 104512, 1:100), Foxp3 (clone MF-14, Biolegend, cat#: 126408, 1:100), CD279 (PD-1) (clone 29F.1A12, BioLegend, cat#: 135231, 1:100), IFN-γ (clone XMG 1.2, Biolegend, cat#:505838, 1:100), Granzyme B (clone QA16A02, Biolegend, cat#:372206, 1:100), BCL6 (clone K112-91, BD Biosciences, cat#: 561522, 1:20), CD8α (clone 53-6.7, Biolegend, 100712, 3ug/mouse), HRP labeled goat anti-mouse IgG secondary

antibody (SouthernBiotech, 1031-05). The following antibodies were used for immunofluorescence studies: Anti-IgD_Biotin (clone: 11-26c, eBioscience, Cat:13-5993-82, 1:200), anti- BCL6_PE (clone: K112-91, BD Biosciences, Cat: 561522, 1:20), anti-IgD_A488 (clone: 11-26c, SouthernBiotech, Cat: 1020-30, 1:200), anti-CD4_BV421 (clone: GK1.5, BioLegend, Cat: 100438, 1:200), anti-CD35_Biotin (clone: 8C12, BD Biosciences, Cat: 553816, 1:100), Streptavidin_A555 (Invitrogen, Cat: S32355, 1:100)

The following antibodies were used in the human study:
CD45 (Biolegend, Cat#: 304024, clone HI30, 1:100), CD3 (Biolegend, Cat#: 300328, clone HIT3a, 1:100), CD19 (Biolegend, Cat#: 392506, clone 4G7, 1:100), CD8 (BD Biosciences, Cat#: 564912, clone SK1, 1:100), CD4 (BioLegend, Cat#: 300536, clone RPA-T4, 1:100), CD38 (Biolegend, Cat#:356606, clone HB-7, 1:100), CD27 (Biolegend, Cat#:124216, clone LG.3A10, 1:100). HRP labeled goat anti-human IgA and IgM secondary antibody (SouthernBiotech, IgA cat#2050-05, IgM cat#2020-05).

Validation

All antibodies are commercially available and have been validated by the manufacturer for use in all the studies described in the paper.
Biolegend:https://www.biolegend.com/en-us/quality/product-development
BD Bioscience:https://www.biocompare.com/Antibody-Manufacturing/355107-Antibody-Manufacturing-Perspectives-BD-Bioscience/
Thermofisher: https://www.thermofisher.com/us/en/home/life-science/antibodies/invitrogen-antibody-validation.html
Please consult the corresponding catalog numbers provided above for the manufacturer validation data on their respective websites.

Animals and other organisms

Policy information about studies involving animals; ARRIVE guidelines recommended for reporting animal research

Laboratory animals

C57BL/6, female, 8-12 weeks-old were used for all the studies.

Wild animals

No wild animals were used.

Field-collected samples

No field-collected samples were used.

Ethics oversight

All the animals were cared in Stanford Animal Facility under specific pathogen-free conditions, 12 light/12 dark cycle, temperatures of ~18-23°C with 40-60% humidity. The study protocol was reviewed and approved by the University Administrative Panel on Laboratory Animal Care.

Note that full information on the approval of the study protocol must also be provided in the manuscript.

Human research participants

Policy information about studies involving human research participants

Population characteristics

Children undergoing tonsillectomy were recruited with IRB approval of donating tissues to this study- 80% for obstructive sleep apnea, 17% for recurrent tonsillitis, and 3% for sleep-discorded breathing. Patient ages ranged from 2-16 years. 44% of participants were female, and 56% are male. Adults (>18 years old, male and female, all ethnic backgrounds)undergoing a surgical procedure that would involve the discarded lymphoid tissue for a spectrum of clinical presentations (e.g. otolaryngology patients undergoing tonsillectomy for sleep apnea and/or cardiothoracic patients undergoing thymectomy, etc.).

Recruitment

Patients were recruited immediately prior to surgery. Patients or their guardians who could not provide explicit written informed consent were excluded. Tonsil patients with serious infections or who were taking systemic immunomodulatory drugs were excluded from the study. All living participants in this study provided written informed consent (or through their guardians). Individuals responsible for consenting patients and collecting tissues were not involved in the scientific aspects of the study. We do not expect any systematic bias in our collection strategy.

Ethics oversight

IRB for Stanford University (tonsils; written informed consent from patients or their legal guardians; approval number 30837 and 60741).

Note that full information on the approval of the study protocol must also be provided in the manuscript.

Flow Cytometry

Plots

Confirm that:

☒ The axis labels state the marker and fluorochrome used (e.g. CD4-FITC).

☒ The axis scales are clearly visible. Include numbers along axes only for bottom left plot of group (a 'group' is an analysis of identical markers).

☒ All plots are contour plots with outliers or pseudocolor plots.

☒ A numerical value for number of cells or percentage (with statistics) is provided.

Methodology

Sample preparation

For murine studies, lymph nodes and lung tissues were mechanically digested, filtered into single cell suspensions, and

Sample preparation	stained using antibodies described above. For human tonsil tissue processing and preparation, it's extensively described in the methods.
Instrument	LSR II (4-laser system, BD)
Software	FACS DIVA (BD)
Cell population abundance	At least 500,000 total events were collected for all FACS analyses.
Gating strategy	Cells were identified based on scatter properties, singlets based on FSC-H VS FSC-A, live cells, CD45+ vs CD45- cells and lymphocytes populations based on established markers shown.

☒ Tick this box to confirm that a figure exemplifying the gating strategy is provided in the Supplementary Information.
